# Progranulin depletion inhibits proliferation via the transforming growth factor beta/SMAD family member 2 signaling axis in Kasumi-1 cells

**DOI:** 10.1016/j.heliyon.2020.e05849

**Published:** 2021-01-08

**Authors:** Kuniaki Yabe, Yasuko Yamamoto, Masao Takemura, Takeshi Hara, Hisashi Tsurumi, Ginette Serrero, Toshitaka Nabeshima, Kuniaki Saito

**Affiliations:** a Department of Disease Control and Prevention, Fujita Health University Graduate School of Health Sciences, Aichi, Japan; b A&T corporation, Kanagawa, Japan; c Advanced Diagnostic System Research Laboratory, Fujita Health University, Graduate School of Health Sciences, Aichi, Japan; d Department of Hematology, Matsunami General Hospital, Gifu, Japan; e University of Maryland Marlene and Stewart Greenebaum Comprehensive Cancer Center, Baltimore, MD, USA; f A&G Pharmaceutical, Inc., Columbia, MD, USA; g Japanese Drug Organization of Appropriate Use and Research, Nagoya, 468-0069, Japan; h Human Health Sciences, Graduate School of Medicine and Faculty of Medicine, Kyoto University, Kyoto, Japan

**Keywords:** Hematologic neoplasms, Progranulin, Mechanistic target of rapamycin complex 1, Transforming growth factor beta

## Abstract

Progranulin is an autocrine growth factor that promotes proliferation, migration, invasion, and chemoresistance of various cancer cells. These mechanisms mainly depend on the protein kinase B (Akt)/mechanistic target of rapamycin (mTOR) pathway. Recent studies have shown that patients with hematopoietic cancer have elevated serum progranulin levels. Thus, the current study aimed to investigate the role of progranulin in hematopoietic cancer cells and how it modulates their proliferation. Both knockdown of progranulin and progranulin neutralizing antibody treatment inhibited proliferation in several human hematopoietic cancer cell lines. Moreover, progranulin depletion not only decreases the phosphorylation level of the Akt/mTOR pathway but also, surprisingly, increases the expression of transforming growth factor-beta (TGF-β) and phosphorylation of mothers against decapentaplegic homolog 2 (SMAD2) in Kasumi-1 cell. Furthermore, LY2109761, an inhibitor of TGF-β receptor type I/II kinase, and TGF-β neutralizing antibody blocked the inhibition of proliferation induced by progranulin depletion. These data provide new insights that progranulin alters cell proliferation via the TGF-β axis and progranulin could be a new therapeutic target for hematopoietic cancers.

## 1 Introduction

Hematopoietic malignancies are types of cancer arising from hematopoietic cells, which are found in the blood, bone marrow, lymph nodes, and spleen. Leukemia, lymphoma, and multiple myeloma are major hematopoietic malignancies. Chemotherapy, molecular targeted therapy, and radiation therapy are effective therapies for these diseases. However, some patients with these diseases will present with relapse and drug resistance, resulting in poor prognosis.

Progranulin, also known as GP88, granulin–epithelin precursor, acrogranin, or PC-cell derived growth factor, is a multifunctional growth factor containing 7.5 repeats of a double cysteine-rich motif [1]. Moreover, it is involved in the proliferation, migration, and invasion of cancer cells and chemoresistance in breast, bladder, colorectal, ovarian, cervical, and hepatocellular cancer and malignant melanoma [2, 3, 4, 5, 6, 7, 8, 9]. Some patients with breast, lung, prostate, and ovarian cancer, glioblastoma, chronic lymphocytic leukemia, and malignant lymphoma have elevated serum progranulin level, which is associated with decreased overall survival, disease-free survival, relapse-free survival, and progression-free survival [10, 11, 12, 13, 14, 15,16, 17]. In hematopoietic malignancy, patients with acute myeloid leukemia who reached complete remission showed significantly decreased plasma progranulin levels after treatment, although there were no changes in plasma progranulin levels among patients who did not achieve complete remission [17]. Therefore, progranulin could be a useful independent prognostic marker for these hematologic malignancies.

As previously mentioned, progranulin is a promising candidate not only as a biomarker of cancer but also as an important molecular target for cancer treatment. Previous studies have revealed that progranulin increases the phosphorylation of proteins in protein kinase B (Akt)/mechanistic target of rapamycin (mTOR) pathway and extracellular signal-regulated kinases (ERK) protein. More specifically, progranulin also protects bladder cancer cells from cisplatin, and promotes proliferation of bladder cancer cells and hepatocellular carcinoma cells via Akt, mTOR, and ERK protein [7, 9, 18]. On the other hand, rapamycin, a mTOR inhibitor, GDC-0941, a phosphoinositide 3-kinase inhibitor, and PD-98059, an ERK inhibitor, partially inhibit cell proliferation and autocrine progranulin expression in ovarian clear cell carcinoma [5]. In hematopoietic malignancies, recombinant progranulin promotes dexamethasone resistance via phosphorylation of Akt and ERK proteins [19]. In particular, recent studies have shown that EPH receptor A2 (EphA2) and drebrin, an F-actin binding protein, play essential roles in progranulin derived, Akt/ERK proteins mediated signal transduction in bladder cancer [18, 20]. Recent findings indicate that the effect of progranulin is mainly dependent on these pathways and some other pathways are not yet fully identified in progranulin related oncogenic signal transduction.

In the present, specific receptors and mediators for progranulin remain controversial. Although tumor necrosis factor receptor (TNFR) has been reported as a receptor for progranulin, other groups have failed to reproduce this progranulin-TNFR binding [21, 22, 23]. Furthermore, sortilin in breast cancer cells, cation-independent mannose 6-phosphate receptor in neuroblastoma cells, and EphA2 in human umbilical vein endothelial cells and bladder cancer cells have been reported to bind extracellular progranulin [24, 25, 26].

We investigated whether blocking progranulin expression inhibits the cell proliferation and examined action mechanism of progranulin on proliferation of hematopoietic cancer cell lines. Our study suggests that progranulin depletion inhibits cell proliferation in malignant hematopoietic cell lines via not only inhibition of the Akt/mTOR pathway, but also via enhancement of the production of transforming growth factor-beta (TGF-β) by using knockdown technology and neutralizing antibodies.

## 2 Material and methods

### 2.1 Cell culture

All cells were purchased from JCRB Cell Bank. The cells were cultured in RPMI1640 medium (FUJIFILM Wako Pure Chemical Corporation, Osaka, Japan) containing 10% fetal bovine serum (Biowest, Nuaillé, France), penicillin (100 IU/mL), and streptomycin (100 μg/mL) (FUJIFILM Wako Pure Chemical, Osaka, Japan).

### 2.2 Transfection

All cells were transfected with the Neon Transfection System (Thermo Fisher Scientific, MA, USA) based on the manufacturer's instructions. The cells were transfected with Validated MISSION siRNA (200 nM) as progranulin-specific siRNA, or MISSION siRNA Universal Negative control (Sigma-Aldrich, MO, USA) as control. They were collected at the indicated times by pipetting into conical tubes. The cells were washed twice with phosphate-buffered solution (PBS) by spinning for 5 min, at 300 RCF, at 4 °C. The cell pellet was lysed with Laemmli buffer supplemented with Protease Inhibitor Cocktail and Phosphatase Inhibitor Cocktail (Nacalai Tesque, Kyoto, Japan) followed by sonication on ice. After centrifugation for 5 min, at 12000 RCF, at 4 °C, the supernatants were collected and stored at −30 °C.

### 2.3 Proliferation assay

The progranulin siRNA or control siRNA-transfected cells were seeded in 96 well plates at a density of 3.5 × 10ˆ5 cells/mL. Proliferation was determined using the CellTiter 96 Non-Radioactive Cell Proliferation Assay (Promega, WI, USA). Anti-human progranulin mouse monoclonal antibody 6B3 was kindly provided from Dr. Ginette Serrero (A&G Pharmaceutical, MD, USA). Normal mouse IgG (FUJIFILM Wako Pure Chemical Corporation, Osaka, Japan) was used as control. The cells were seeded in 96 well plates at the density of 5.0 × 10ˆ4 cells/mL with anti-progranulin antibody or normal mouse IgG (200 μg/mL), and proliferation was determined based on the above-mentioned description. To regulate the signaling pathway, the cells were cultured with the combination of rapamycin (20 nM) (FUJIFILM Wako Pure Chemical, Osaka, Japan), chloroquine (20 nM), Z-VAD-FMK (50 μM) (MBL, Aichi, Japan), LY2109761 (250 μM) (ChemScene, NJ, USA), or anti-TGF-β antibody (2 μg/mL) (R&D Systems, MN, USA).

### 2.4 Western blotting

Whole cell lysate was quantified using BCA assay, and each sample was separated via electrophoresis on 7.5% or 10% sodium dodecyl sulfate-polyacrylamide gels. Proteins were then transferred to PVDF membrane, which was blocked in 5% skim milk in TBST (Tris buffer saline with 0.1 % v/v Tween-20) at room temperature for 60 min. After washing with TBST buffer, the membrane was incubated with anti-progranulin (kind gift from Dr. Ginette Serrero; A&G Pharmaceutical, MD, USA), phospho-mTOR, total-mTOR, phospho-Akt, total Akt, phospho-ERK, total-ERK, cleaved PARP, caspase-3, Bax, Bcl-2, XIAP (Cell Signaling Technology, MA, USA), and β-actin (Sigma-Aldrich, MO, USA) antibodies at a concentration of 1.0 μg/mL (anti-progranulin), dilution of 1:15000 (anti-β-actin) or 1:1000 (other antibodies) in Can Get Signal (TOYOBO, Osaka, Japan) at 4 °C overnight. On the following day, the membrane was washed with TBST buffer and was incubated with horseradish peroxidase conjugated anti-mouse IgG antibody or horseradish peroxidase conjugated anti-rabbit IgG antibody (Jackson ImmunoResearch, PA, USA) at a dilution of 1:50000 in Can Get Signal at room temperature for 60 min followed by ECL prime (GE Healthcare, IL, USA). The protein bands on the membrane were detected using a CCD camera system (ATTO, Tokyo, Japan). Densitometric analysis of each protein signal was performed using the CS Analyzer 4 (ATTO, Tokyo, Japan).

### 2.5 Quantification of extracellular progranulin and TGF-β

Kasumi-1 cells were seeded after transfected progranulin-specific siRNA in 6 well plates at a density of 3.5 × 10ˆ5 cells/mL. After 48 h, they were collected by pipetting into conical tubes and centrifuged for 5 min, at 300 RCF, at 4 °C. Then, the supernatant was transferred into new tubes and stored at -80 °C. The extracellular progranulin concentration was determined by using human progranulin sandwich enzyme-linked immunosorbent assay (ELISA) kit (A&G Pharmaceutical, MD, USA). The extracellular TGF-β concentration was determined by using Simple Plex Ella (ProteinSimple, CA, USA).

### 2.6 Quantification of cell apoptosis

Kasumi-1 cells were seeded after transfected progranulin-specific siRNA in 6 well plates at a density of 3.5 × 10ˆ5 cells/mL. After 48 h, Kasumi-1 cells were transferred into conical tubes and stained with Annexin V and 7-Aminoactinomycin D to quantify cell apoptosis by using Guava Muse cell analyzer and Annexin V & Dead Cell Kit (Luminex, TX, USA).

### 2.7 RNA extraction, RT-qPCR and mRNA microarray

The cells were cultured in RPMI 1640 medium containing 10% fetal bovine serum, penicillin (100 IU/mL), and streptomycin (100 μg/mL) with anti-progranulin antibody (200 μg/mL) (A&G Pharmaceutical, MD, USA) or normal mouse IgG, used as control, for 8 h. Then, RNA was extracted using the RNeasy Mini Kit (QIAGEN, Hilden, Germany) based on the manufacturer's instructions. In the mRNA microarray analysis, procedures for reverse transcription, labeling, and microarray experiments were performed using Clariom D assay for human samples (Thermo Fisher Scientific, MA, USA) by Filgen Inc. (Aichi, Japan). A microarray data analysis tool (Filgen, Aichi, Japan) was used to identify differentially expressed pathways and genes. In the RT-qPCR analysis, reverse transcription and qPCR were performed using the PrimeScript RT Reagent Kit (TaKaRa Bio, Shiga, Japan) and PowerUp SYBR Green Master Mix (Applied Biosystems, CA, USA) based on the manufacturer's instruction. According to a previous report, the following primer sequences were used in qPCR: TGF-β receptor type II forward, AGATACATGGCTCCAGAAGTCC; TGF-β receptor type II reverse, ACTTCTCCCACTGCATTACAGC [27]; GAPDH forward, GGAGCGAGATCC CTCCAAAAT; GAPDH reverse, GGCTGTTGTCATACTTCTCATGG [28].

### 2.8 Statistical analysis

All statistical analyses were performed using GraphPad Prism 6 (GraphPad Software, CA, USA). Results from experiments with two groups was analyzed by using two-tailed Student's *t* test. Results from experiments with more than two groups was analyzed by using Tukey's test or Dunnett's test.

## 3 Results

### 3.1 Progranulin knockdown and progranulin neutralizing antibody inhibit cell proliferation in malignant hematopoietic cell lines

**Figure 1:**
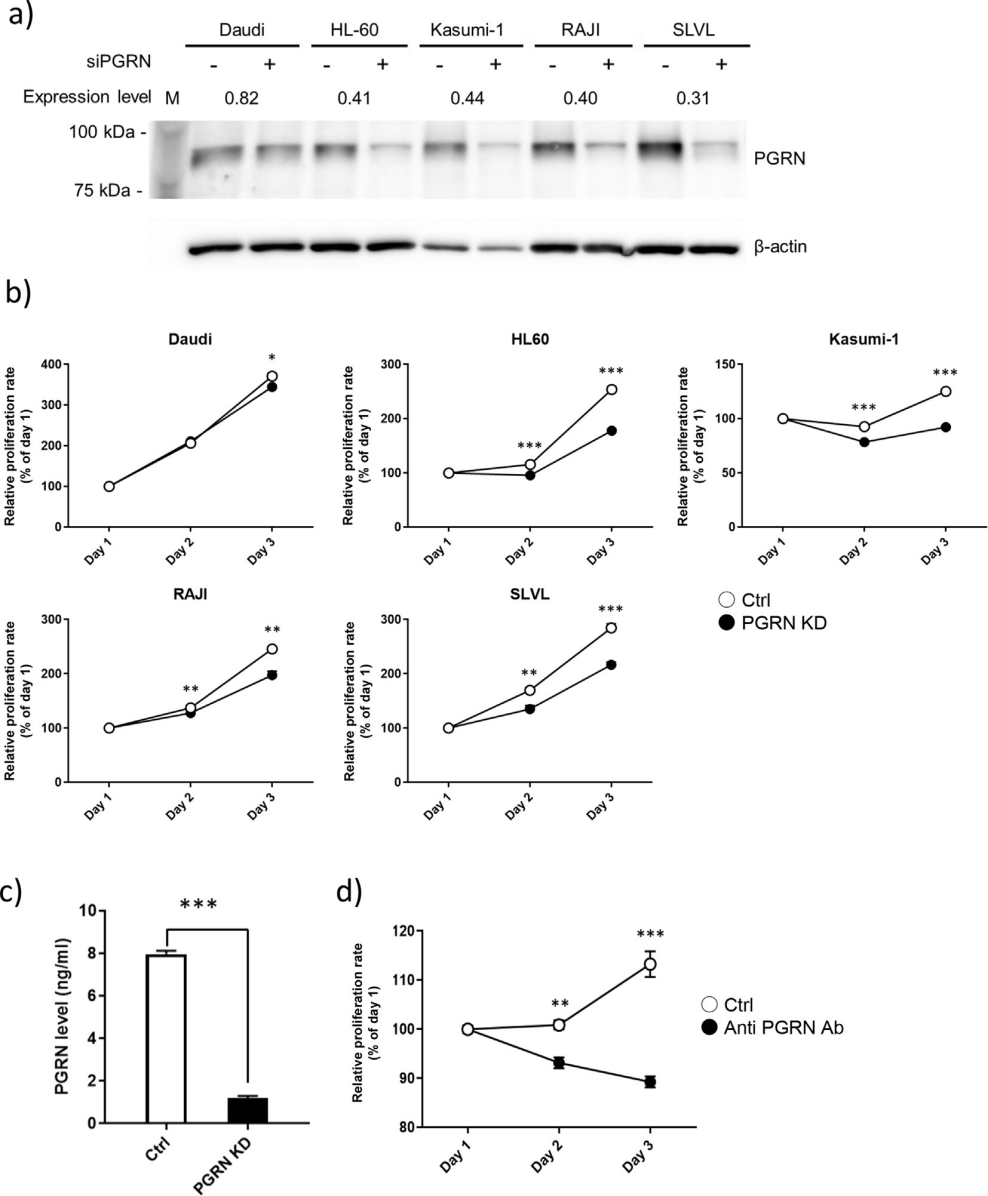
Progranulin knockdown and progranulin neutralizing antibody inhibit cell proliferation in malignant hematopoietic cell lines. a) Hematopoietic cancer cell lines were transfected with progranulin specific siRNA or control siRNA. Whole cell lysate was collected 24 h after transfection and expression level of Progranulin in the treated cells was analyzed by Western blotting. Expression level of progranulin was normalized to that of β-actin and knock down efficiency was calculated. *n* = 3. b) Proliferation of treated cell was determined by MTT assay. Error bars indicate the SD from mean; *n* = 3. (∗*P* < 0.05, ∗∗*P* < 0.01, ∗∗∗*P* < 0.001; two-tailed Student's *t* test) c) Kasumi-1 was transfected progranulin specific siRNA and control siRNA. The supernatant was collected 48 h after transfection and extracellular progranulin level was determined by sandwich ELISA. Error bars indicate the SD from mean; *n* = 3. (∗∗∗*P* < 0.001; two-tailed Student's *t* test) d) Kasumi-1 was cultured with anti progranulin antibody (200 μg/ml) or control antibody and proliferation of treated cells was determined by MTT assay. Error bars indicate the SD from mean; *n* = 3. (∗∗*P* < 0.01, ∗∗∗*P* < 0.001; two-tailed Student's *t* test).

Because over 80% of newly diagnosed patients with hematopoietic malignancies suffer from lymphoma and leukemia [29], cell lines such as Daudi (Burkitt lymphoma), HL60 (acute promyelocytic leukemia), Kasumi-1 (acute myeloid leukemia), RAJI (Burkitt lymphoma), and SLVL (splenic B cell lymphoma) were used to investigate the role of progranulin in the proliferation of human hematopoietic cancer cells. First, progranulin-specific siRNA was transfected into these cell lines. The inhibition of progranulin expression was confirmed by Western blotting (Figure 1a). The relative expression levels of progranulin in all cell lines were decreased to about 40% of the control siRNA-transfected cells, but not Daudi.

Next, the effect of the progranulin knockdown on cell proliferation was examined. As expected, the proliferation of all cell lines was significantly inhibited by the siRNA-mediated knock down of progranulin. The relative proliferation levels at the day 3 were 92.9% (Daudi), 70.1% (HL60), 73.6% (Kasumi-1), 80.4% (RAJI), and 86.1% (SLVL) compared to the control groups (Figure 1b). Currently, the standard care for patients with acute promyelocytic leukemia, origin of HL60 cells, is chemotherapy which results in good outcome [30]. Although few molecular-targeted therapies are available for acute myeloid leukemia, origin of Kasumi-1 cells, the complete remission rate is only 40–50% in newly diagnosed patients [31]. Therefore, it is important to provide information about novel targets for the therapy of acute myeloid leukemia. Next, we investigated whether extracellular progranulin level was decreased in Kasumi-1 cells transfected progranulin-specific siRNA, since progranulin is an autocrine growth factor also found in extracellular fluids, including serum.

As shown in Figure 1c, the extracellular progranulin level was decreased by progranulin-specific siRNA transfection. Moreover, the proliferation of Kasumi-1 cells was significantly inhibited by treatment with anti-progranulin antibody (Figure 1d). Interestingly, the antibody showed higher inhibitory effect on the proliferation of Kasumi-1 cells than that of knock down by siRNA transfection. Although siRNA may suppress the majority of progranulin expression, some of progranulin remains in the cells and in the culture medium, it may increase cell proliferation. These results obtained with two independent progranulin depletions, by siRNA and neutralizing antibody, strongly indicate that progranulin depletion could be one of the new strategies for hematopoietic cancers.

Further, these results suggest that progranulin plays a role in the proliferation of malignant hematopoietic cell lines similarly to other types of cancer cell lines, such as MCF-7, SKBR-3, RPMI8226, HeLa, H8, KKU-100, and HepG2 [2, 27, 32, 33, 34, 35].

### 3.2 Progranulin depletion decreases the phosphorylation of proteins in the Akt/mTOR pathway

**Figure 2:**
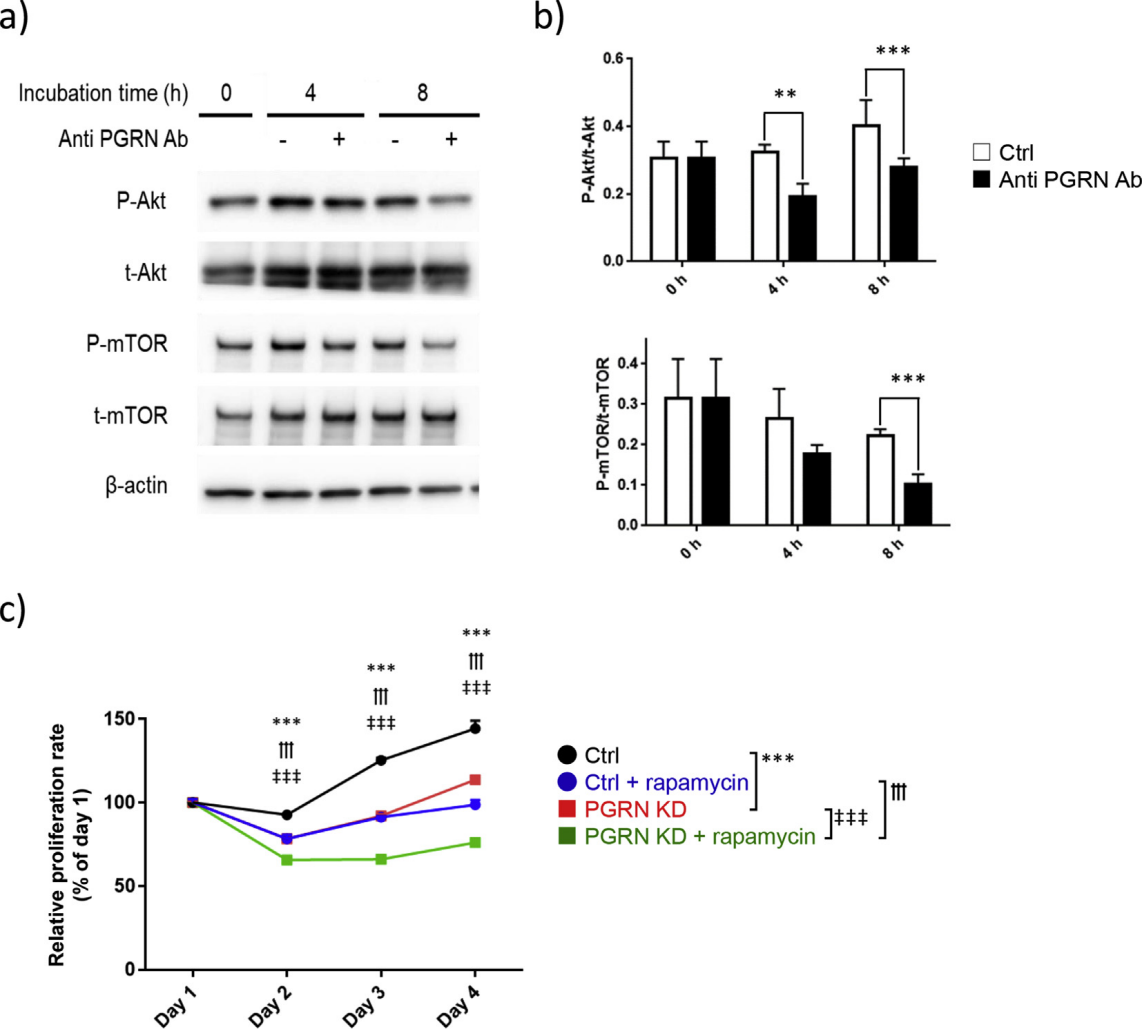
Progranulin depletion decreases the phosphorylation of proteins in the Akt/mTOR pathway. a) Kasumi-1 cells were cultured with anti progranulin antibody (200 μg/ml) or control antibody for the indicated times. Whole cell lysate was collected and expression level of protein was analyzed by western blotting. b) Phosphorylation level of protein was normalized to that of each corresponding total protein. Error bars indicate the SD from mean; *n* = 3. (∗∗*P* < 0.01, ∗∗∗*P* < 0.001; two-tailed Student's *t* test) c) Progranulin specific siRNA or control siRNA transfected Kasumi-1 cells were cultured with rapamycin (20 nM). Proliferation of treated cells was determined by MTT assay. Error bars indicate the SD from mean; *n* = 3. (∗∗∗*P* < 0.001 between Ctrl and PGRN KD, †††*P* < 0.001 between Ctrl + rapamycin and PGRN KD + rapamycin, ‡‡‡*P* < 0.001 between PGRN KD and PGRN KD + rapamycin; Tukey's test).

Previous reports have shown that recombinant progranulin increases the phosphorylation of the proteins in Akt/mTOR pathway. Conversely, progranulin knockdown suppresses the phosphorylation of the proteins in the Akt/mTOR pathway [35]. Additionally, Akt/mTOR pathway is often overactivated in human tumors resulting in tumor growth [36]. Therefore, to examine the role of progranulin in the phosphorylation of the above-mentioned proteins in malignant hematopoietic cells, Kasumi-1 cells were cultured with anti-progranulin antibody and measured phosphorylation of signaling molecules in Akt/mTOR pathway at 0, 4, and 8 h after the treatment. The phosphorylation of the Akt protein was significantly suppressed at 4 and 8 h after the treatment with anti-progranulin antibody. In addition, the phosphorylation of the mTOR protein was significantly inhibited at 8 h (Figures 2a, b). These results suggest that progranulin increases the phosphorylation of the Akt/mTOR signaling pathway in hematopoietic malignant cells.

Next, an association of decreases between phosphorylation of the Akt/mTOR pathway and cell proliferation induced by progranulin depletion was further investigated by using rapamycin, a specific mTOR inhibitor. siRNA-treated Kasumi-1 cells were cultured in the presence of rapamycin. Rapamycin decreased cell proliferation regardless of whether Kasumi-1 cells were treated with control siRNA or progranulin-specific siRNA. Interestingly, the combination of progranulin-specific siRNA with rapamycin significantly reduced cell proliferation compared with that of progranulin-specific siRNA or rapamycin alone (Figure 2c). These data indicate that there is a new mTOR independent progranulin-related pathway in the proliferation of Kasumi-1 cells, and progranulin will be a target of new therapeutic strategy to hematopoietic cancers.

### 3.3 Progranulin depletion induces caspase-independent cell death

**Figure 3:**
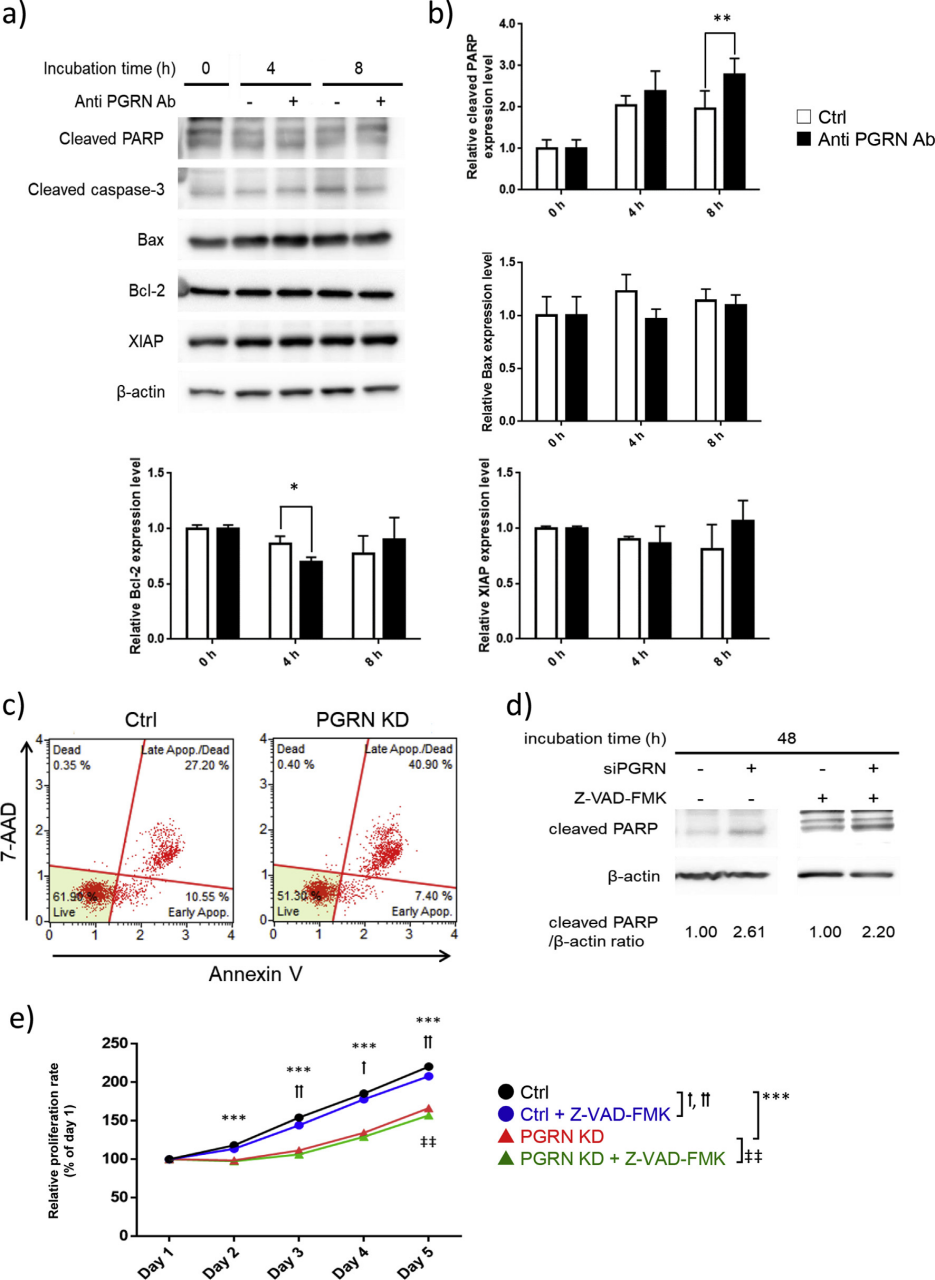
Progranulin depletion induces caspase-independent apoptosis. a) Kasumi-1 cells were cultured with anti progranulin antibody (200 μg/ml) or control antibody for the indicated hours. Whole cell lysate was collected and expression level of canonical apoptosis related proteins was analyzed by western blotting. b) Expression level of apoptosis related proteins was normalized to that of β-actin. Error bars indicate the SD from mean; *n* = 4. (∗*P* < 0.05, ∗∗*P* < 0.01; two-tailed Student's *t* test) c) Kasumi-1 cells transfected progranulin-specific or control siRNA were cultured for 48 h and cell apoptosis was analyzed by using Annexin V & Dead Cell Kit. d) Progranulin specific siRNA or control siRNA transfected Kasumi-1 cells were cultured with or without Z-VAD-FMK (50 μM) for 48 h. Whole cell lysate was collected and PARP cleavage was analyzed by western blotting. e) Progranulin specific siRNA or control siRNA transfected Kasumi-1 cells were cultured with or without Z-VAD-FMK (50 μM) and proliferation of treated cells was determined by MTT assay. Error bars indicate the SD from mean; *n* = 3. (∗∗∗*P* < 0.001 between Ctrl and PGRN KD. †*P* < 0.05, ††*P* < 0.01 between Ctrl and Ctrl + Z-VAD-FMK. ‡‡*P* < 0.01 between PGRN KD and PGRN KD + Z-VAD-FMK; Tukey's test).

Because progranulin also prevents apoptosis in breast and colorectal cancer cells via mTOR independent manner [37, 38], it is conceivable that progranulin depletion causes apoptosis via mTOR independent manner in hematopoietic cancer cells as well. To elucidate the possible role of the progranulin-related signaling pathway in apoptosis, Kasumi-1 cells were cultured with anti-progranulin or control antibody to examine the apoptosis-related proteins by Western blotting. The expression of cleaved poly (ADP-ribose) polymerase (PARP), a marker of apoptosis in the cells, was significantly increased, and BCL2 apoptosis regulator (Bcl-2), an inhibitor of apoptosis, was decreased by the anti-progranulin antibody. However, canonical apoptosis-related proteins, including caspase-3, BCL2-associated X apoptosis regulator (Bax), and X-linked inhibitor of apoptosis (XIAP), were unchanged (Figures 3a, b). Furthermore, Kasumi-1 cells transfected progranulin-specific siRNA also showed an increased proportion of apoptotic cells (Figure 3c). Although these data suggested progranulin depletion induced apoptosis, contribution of caspase-dependent apoptosis pathway was still unclear. To validate the relationship between progranulin depletion and caspase-dependent apoptosis, Kasumi-1 cells transfected progranulin-specific siRNA were cultured in the presence of the pan-caspase inhibitor Z-VAD-FMK for 48 h. In accordance with the results with progranulin neutralizing antibody in Figure 3a and c, progranulin-specific siRNA increased expression of cleaved PARP compared to control siRNA, and the relative expression level of cleaved PARP was not changed by Z-VAD-FMK (Figure 3d). In addition, in these cells, Z-VAD-FMK could not prevent the inhibition of proliferation induced by progranulin depletion (Figure 3e). Although PARP is a representative substrate for caspases which is activated in apoptosis, other molecules, for instance, calpains, cathepsins, and granzymes, may be activated in caspase independent cell death and also can cleave PARP [39, 40]. These results suggest that cell death induced by progranulin depletion is independent from canonical caspase-dependent pathway and dependent on unknown molecules.

### 3.4 Upregulation of the TGF-β signaling axis by progranulin depletion

**Figure 4:**
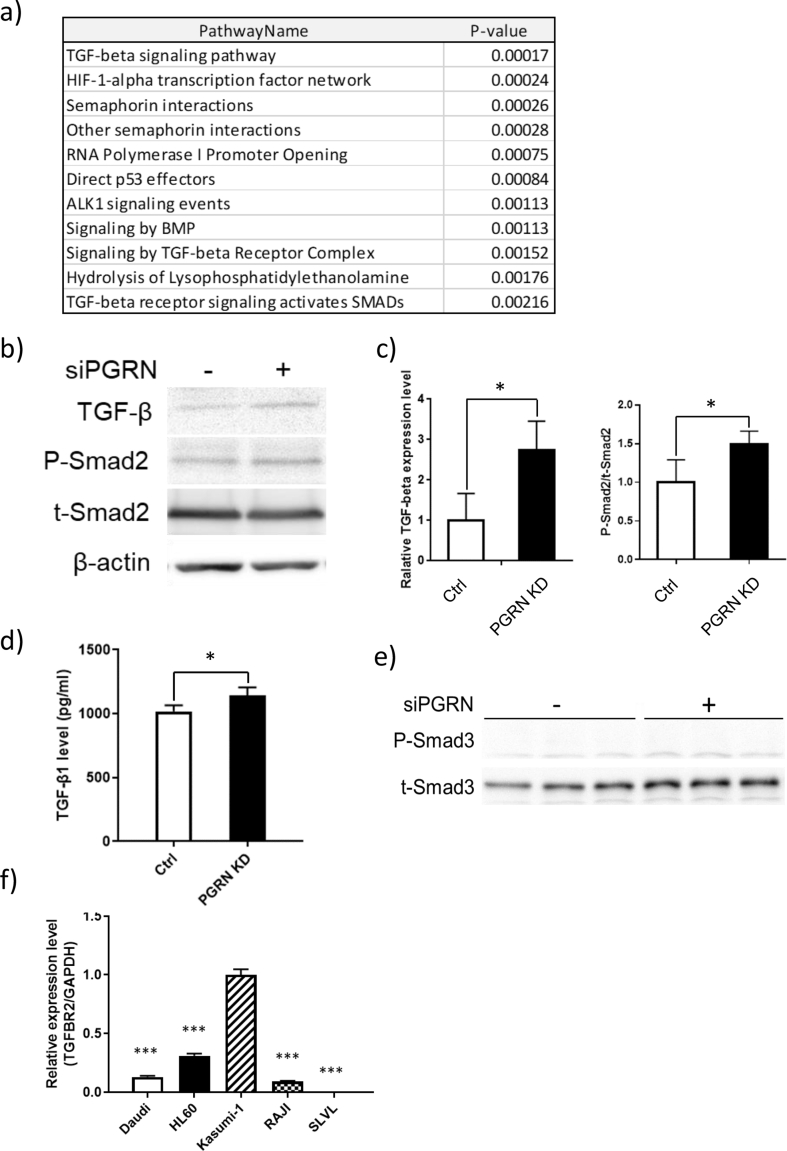
Upregulation of the TGF-β signaling axis by progranulin depletion. a) Kasumi-1 cells were cultured with anti-progranulin antibody (200 μg/ml) or control antibody for 8 h. RNA extraction and mRNA microarray were performed, and significantly upregulated pathways were identified. b) Progranulin specific siRNA or control siRNA transfected Kasumi-1 cells were cultured for 48 h. Whole cell lysate was collected and expression of TGF-β, P-Smad2 were analyzed by western blotting. c) Expression level of TGF-β was normalized to that of β-actin, expression level of P-Smad2 was normalized to that of t-Smad2. Error bars indicate the SD from mean; *n* = 4. (∗*P* < 0.05; two-tailed student's *t* test) d) The supernatant was collected 48 h after transfection and extracellular TGF-β level was determined by Ella Simple Plex. Error bars indicate the SD from mean; *n* = 4. (∗*P* < 0.05; two-tailed Student's *t* test) e) Expression level of P-Smad3 and t-Smad3 in Kasumi-1 cells transfected progranulin-specific siRNA or control siRNA were analyzed by Western blotting. f) Total RNA was extracted from untreated cell lines to perform RT-qPCR. Expression level of TGF-β receptor type II was normalized to that of GAPDH. Error bars indicate the SD from mean; n = 4. (∗∗∗P < 0.001 compared with Kasumi-1; Dunnett's test).

To further explore other possible progranulin-related signaling pathways, a microarray analysis of mRNA expression was conducted. Kasumi-1 cells were cultured with anti-progranulin antibody or control antibody for 8 h, and the total RNA was extracted to perform a comparative mRNA microarray analysis. Interestingly, progranulin inhibition upregulated the mRNA expression of TGF-β super family, hypoxia inducible factor 1 subunit alpha (HIF-1α), and semaphorin pathways (Figure 4a). In these pathways, TGF-β and HIF-1α are well known as tumor related factors. Although HIF-1α is a prognostic factor in breast cancer patients, knockout of HIF-1α in the human chronic myeloid leukemia K562 cells, showed no effect on proliferation [41, 42, 43]. Interestingly, TGF-β neutralizing antibodies enhanced the proliferation of myeloid leukemia cells [44]. Moreover, TGF-β transfected HL60 cells reduced xenograft growth in nude mice [45]. Thus, further analysis was focused on the effect of progranulin on TGF-β signaling pathway.

Kasumi-1 cells transfected with progranulin specific siRNA were cultured for 48 h, and the expression of TGF-β-related proteins was analyzed by Western blotting. As shown in Figures 4b and c, the expression of TGF-β and the phosphorylation of mothers against decapentaplegic homolog 2 (SMAD2), a downstream molecule of TGF-β receptor, were increased when progranulin expression was inhibited. In accordance, extracellular TGF-β concentration in Kasumi-1 cells transfected progranulin-specific siRNA was also increased (Figure 4d). Although mothers against decapentaplegic homolog 3 (SMAD3), another downstream molecule of TGF-β receptor, was also examined, phosphorylated Smad3 was not increased by progranulin-specific siRNA (Figure 4e). Since we used two different approaches of progranulin neutralization (Figure 4a) and knockdown (Figures 4b, c, d) to show TGF-β upregulation induced by inhibition of progranulin pathway in Kasumi-1 cells, it indicates that increase of TGF-β expression by progranulin depletion is specific and reproducible.

TGF-β induces cell cycle arrest, differentiation and apoptosis. Since proliferation of cancer cells is negatively influenced by these effects, down regulation of TGF-β receptor type II (TGFBRII) in cancer cells is often observed [46]. To confirm the expression of TGFBRII, mRNA was extracted from untreated Daudi, HL60, Kasumi-1, RAJI, and SLVL cells to perform quantitative PCR. As shown in Figure 4f, normalized expression levels of TGFBRII were widely varied among different cells. Importantly, although SLVL cells have relatively low expression level of TGFBRII compared to Kasumi-1 (0.005 vs 1.000), SLVL cells also showed high sensitivity to depletion of progranulin (Figure 1b). These results suggest the molecules affected by progranulin depletion may be different between cell to cell. In accordance with the above hypothesis, in SLVL cells, the combination of progranulin-specific siRNA and rapamycin failed to show an additive inhibitory effect compared to rapamycin alone (Supplementary Figure 1).

**Figure 5:**
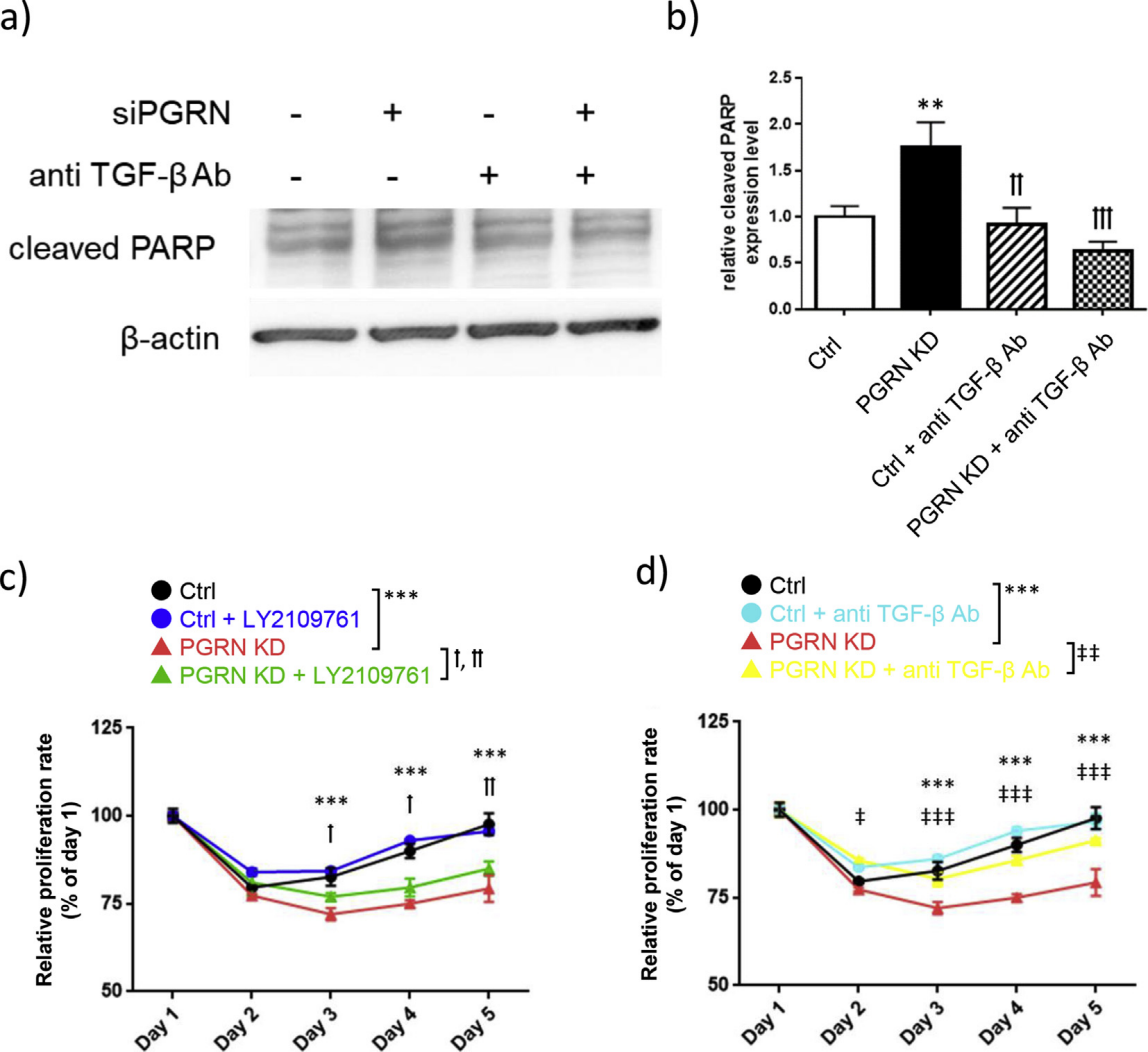
Blocking of the TGF-β signaling axis attenuates inhibition of cell proliferation by progranulin depletion. a) Kasumi-1 cells transfected progranulin-specific siRNA or control siRNA were cultured with or without anti TGF-β antibody (2 μg/ml) for 48 h. Whole cell lysate was collected and expression of cleaved PARP was analyzed by Western blotting. b) Expression level of cleaved PARP was normalized to that of β-actin. Error bars indicate the SD from mean; n = 4. (∗∗P < 0.01 compared with ctrl. ††P < 0.01, †††P < 0.001 compared with PGRN KD; Tukey's test) c) Progranulin specific siRNA or control siRNA transfected Kasumi-1 cells were cultured with the combination of LY2109761 (250 μM). d) or anti TGF-β antibody (2 μg/ml). Proliferation of treated cells was determined by MTT assay. Error bars indicate the SD from mean; *n* = 3. (∗∗∗*P* < 0.001 between Ctrl and PGRN KD. †*P* < 0.05, ††*P* < 0.01 between PGRN KD and PGRN KD + LY2109761. ‡*P* < 0.05, ‡‡‡*P* < 0.001 between PGRN KD and PGRN KD + TGF-β Ab; Tukey's test).

TGF-β inhibits the proliferation of myeloid leukemia, origins of Kasumi-1 cells [47]. To evaluate whether TGF-β inhibits the proliferation of Kasumi-1 cells, the expression level of cleaved PARP was analyzed in Kasumi-1 cells transfected with progranulin-specific siRNA in the presence or absence of anti-TGF-β antibody. Anti-TGF-β antibody treatment decreased the increase of expression of cleaved PARP induced by progranulin depletion to the level of control siRNA transfected cells (Figures 5a, b).

Finally, to confirm the role of TGF-β in the inhibition of proliferation induced by progranulin depletion, we investigated whether treatment TGF-β receptor type I kinase inhibitor LY2109761 or anti-TGF-β antibody could block the effect of progranulin depletion. Progranulin depletion significantly decreased proliferation of Kasumi-1 cells (Figure 5c, d). Either anti-TGF-β antibody or LY2109761 partly blocked the inhibition of proliferation induced by progranulin depletion. This result suggests that the inhibition of proliferation induced by progranulin depletion is partly dependent on the TGF-β signaling axis (Figure 5).

## 4 Discussion

The goal of this study was to explore the role of Akt/mTOR independent progranulin related signaling pathway in regulation to the proliferation of hematopoietic cancer cells. First of all, by using molecular and immunological approaches, whether progranulin depletion inhibits the proliferation of hematopoietic cancer cells was investigated. The proliferation of HL60 and Kasumi-1 cells was strongly inhibited by progranulin knock down compared to other cells, although there was no difference in the efficiency of progranulin knockdown among them except for Daudi cells.

In RAJI cells (Burkitt lymphoma) and SLVL cells (splenic B cell lymphoma), mutations of DNA-binding protein inhibitor ID-3 (ID3), transcription factor 3 (TCF3), and neurogenic locus notch homolog protein 2 (NOTCH2) increase phosphorylation of proteins in Akt/mTOR pathway [48, 49, 50]. Therefore, it is likely that positive feedback loops in Akt/mTOR pathway compensate for the suppression of Akt/mTOR signal by depletion of progranulin in RAJI cells and SLVL cells.

Depletion of progranulin inhibited the proliferation and phosphorylation of the proteins in the Akt/mTOR signaling pathway in hematopoietic cancer cell lines similarly to other cancer cell types [7]. Moreover, the effect of rapamycin, an inhibitor of mTOR protein, on the proliferation was strengthened by knock down of progranulin. Thus, progranulin regulates proliferation, at least in part, through the mTOR independent signaling pathway in the hematopoietic cancer cells.

To elucidate the mTOR independent progranulin-related signaling pathway, apoptosis signaling was examined because progranulin prevents apoptosis via mTOR independent manner [37, 38]. The inhibition of proliferation induced by progranulin depletion could be explained by caspase-independent cell death. In neuroblast cells, overexpression of progranulin decreases expression of cathepsins, while knockdown of progranulin increases expression of that [51]. Moreover, upregulated cathepsins could induce cell death in cancer cells [52]. In agreement with this observation, expression of cathepsin B mRNA was up to 3.9-fold and cathepsin D mRNA was up to 2.5-fold in the microarray analysis (data not shown). These molecules may also contribute to the cell death induced by progranulin depletion.

Interestingly, the microarray and Western blotting experiments uncovered that TGF-β was overexpressed by progranulin depletion in the cells treated with both anti-progranulin antibody and siRNA. Furthermore, both TGF-β neutralizing antibody and LY2109761, TGF-β receptor kinase inhibitor, blocked the inhibitory effect on proliferation induced by progranulin depletion. Because cytotoxicity was observed in Kasumi-1 cells when cultured with higher dose of LY2109761 than 250 μM (data not shown), it might be the reason why LY2109761 (250 μM) showed weaker effects compared to TGF-β neutralizing antibody. This is the first report showing that progranulin depletion induces the production of TGF-β in acute myeloid leukemia cell lines, and suppresses the proliferation of cancer cells via TGF-β as shown in Figure 5. One limitation in this study is why progranulin depletion facilitates TGF-β expression is still unclear. A possible explanation for this question is an involvement of adenosine monophosphate-activated protein kinase1 (AMPK) which expressed ubiquitously to maintain energy homeostasis, Because progranulin increases expression of AMPK in stress conditions and AMPK is also known to be a negative regulator of TGF-β expression and signal transduction, it is conceivable that progranulin depletion induces TGF-β expression via suppression of AMPK [53, 54]. We should confirm this point in the future experiments.

Noteworthy, TGF-β receptor type II (TGFBRII) is highly expressed in Kasumi-1 cells compared to other cell lines. Loss of function of TGFBRII via down regulation of transcription level or somatic mutation has been reported as a mechanism to evade effect of TGF-β in cancer cells [46]. SLVL cells showed very low level of TGFBRII expression in this study, and in these cells, the inhibitory effect of the combination of progranulin depletion and rapamycin was almost equal to rapamycin alone. This result suggests that inhibitory molecular mechanisms induced by progranulin depletion are categorized into at least two types, such as 1) both Akt/mTOR and TGF-β pathways in Kasumi-1 cells, 2) Akt/mTOR pathway alone in SLVL cells. Additionally, the microarray analysis revealed a 2.3-fold and 1.7-fold changes of mRNA expression of TGF-β receptor type I and II, respectively, in Kasumi-1 cells treated with progranulin-specific antibody (data not shown). This result suggests that TGF-β signal transduction produced by progranulin depletion may form a positive regulation feedback.

Treatment with progranulin-specific antibody also decreased phosphorylation of ERK protein in Kasumi-1 cells (Supplementary Figure 2). In acute myeloid leukemia cells, origins of Kasumi-1 cells, the suppression of Akt/mTOR pathway induces activation of ERK pathway to compensate for pro-survival signal transduction [55]. Taken together, these results suggest that the role of Akt/mTOR and ERK pathways in progranulin related signal transduction is still important in hematopoietic cancer cells, and progranulin is one of the attractive targets to suppress both pathways simultaneously.

In this study, we showed that progranulin depletion also induced TGF-β production resulting in inhibition of cancer cell proliferation. Previous report supports our results that in progranulin knockout mice after traumatic injury, microglia increases TGF-β expression level and upregulates Smad3 signaling pathway [56]. In contrast, exogenous progranulin enhances TGF-β production in human CD4^+^ T cells [57].

The reason of the discrepancy in functions of progranulin can be attributed to variation in progranulin receptors. Tumor necrosis factor receptor (TNFR) in chondrocytes, sortilin in breast cancer cells, cation-independent mannose 6-phosphate receptor in neuroblastoma cells, and EphA2 in human umbilical vein endothelial cells and bladder cancer cells also bind extracellular progranulin [21, 22, 24, 25, 26]. Especially, EphA2 and drebrin, an F-actin binding protein, play an essential roles for progranulin derived cancer promoting functions via phosphorylation of Akt and ERK proteins in bladder cancer [20]. However, the microarray analysis revealed mRNA expression level of EphA2 and drebrin in Kasumi-1 cells were undetectable and unchanged regardless of treatment with progranulin-specific antibody (data not shown). Thus, the identity of the major receptors for progranulin is still not completely established and the diversity of receptors in various cell types may modify the physiological functions of progranulin.

Although several mTOR inhibitors have been developed because of the importance of Akt/mTOR pathway in cancer cell proliferation and chemoresistance, cancer cells frequently become mTOR inhibitor resistant. mTOR inhibitor generally inhibits cancer cell proliferation via cell cycle arrest but does not induce cell death, which leads to the selective pressure to acquire mTOR inhibitor resistance [58]. Considering the properties of progranulin, anti-progranulin antibody has several advantages for cancer therapy. Firstly, progranulin depletion not only downregulates Akt/mTOR signaling pathway but also upregulates production of TGF-β in cancer cells. As a result, progranulin depletion triggers cell death, it could solve the problem of resistance to mTOR inhibitor in cancer cells by upregulation of TGF-β production.

In hematopoietic malignancy, reduced plasma TGF-β levels were observed in patients, while bone marrow plasma concentrations of TGF-β returned to normal after complete remission [59, 60]. It suggests that reduction in TGF-β might be a risk in patients with hematopoietic malignancies. On the contrary, production of TGF-β might promote tumor growth [61]. Thus, the effect of TGF-β in progranulin neutralizing is a double-edge sword which can inhibit or promote the proliferation of cancer cells.

In TGF-β signaling pathway, TGF-β phosphorylates Smad2 and Smad3 to induce cell cycle arrest. Concomitantly, Rac family small GTPase 1 (Rac1) expression is induced in TGF-β treated cells to counteract this TGF-β mediated cytostasis [62]. Notably, since expression level of Rac1 mRNA was also increased up to 1.9-fold in the microarray analysis after neutralizing progranulin (data not shown), upregulated Rac1 might block cell death induced by depletion of progranulin. Therefore, these data raise the possibility that Rac1 inhibitor, like NSC23766, may enhance the antitumor effect of progranulin neutralization.

In terms of Rac-1 inhibitor, R-ketorolac has been approved by FDA which suppresses adhesion, migration, and invasion of ovarian cancer cells [63]. Further, clinical trial of R-ketorolac for ovarian cancer patients as metastasis inhibitor is ongoing (trial ID: NCT02470299). Taken together, Rac1 inhibitor might enhance the antitumor effect of progranulin depletion and suppress metastasis via production of TGF-β.

Additionally, progranulin depletion suppresses transformation of cancer associated fibroblasts (CAFs) [64]. CAFs facilitate tumor growth and metastasis via secretion of growth factor, acquisition of chemoresistance, potentiation of angiogenesis, and downregulation of cell adhesion molecules between cancer cells [65]. In terms of targeting tumor microenvironment, progranulin depletion is also expected to show antitumor effect.

Finally, one limitation in the clinical use of anti-progranulin antibody is the fact that the identity of its signaling receptor in hematopoietic malignancies remains unknown. Because progranulin is a multifunctional molecule in many types of diseases and has multiple receptors depending on the cell types, we should carefully investigate the risks from systemic depletion of progranulin. Only human phase 1 safety and efficacy study of anti-progranulin therapy could address this question.

In summary, this is the first report demonstrating that progranulin depletion inhibits proliferation of hematopoietic cancer cells through TGF-β production and inhibition of Akt/mTOR pathway. Progranulin is a promising new molecular target to develop an effective therapy in hematopoietic malignancies.

## Declarations

### Author contribution statement

Yasuko Yamamoto, Kuniaki Yabe: Conceived and designed the experiments; Performed the experiments; Analyzed and interpreted the data; Wrote the paper.

Masao Takemura: Performed the experiments; Contributed reagents, materials, analysis tools or data.

Takeshi Hara, Hisashi Tsurumi: Contributed reagents, materials, analysis tools or data.

Ginette Serrero: Contributed reagents, materials, analysis tools or data; Wrote the paper.

Toshitaka Nabeshima, Kuniaki Saito: Conceived and designed the experiments; Analyzed and interpreted the data; Wrote the paper.

### Funding statement

This work was supported by A&T corporation, JSPS KAKENHI Grant Numbers 18K19761(KS), 19K07490 (YY) and 17H04252 (TN), the Private University Research Branding Project from the Ministry of Education, Culture, Sports, Science and Technology of Japan (MEXT) and the Smoking Research Foundation (KS).

### Data availability statement

No data was used for the research described in the article.

### Declaration of interests statement

The authors declare the following conflict of interests: Kuniaki Yabe; performed this study while an employee of A&T corporation. Ginette Serrero; performed this study while an employee of A&G Pharmaceutical and is also a shareholder of A&G Pharmaceutical. The remaining authors declare no conflict of interest.

### Additional information

No additional information is available for this paper.

## Appendix A Supplementary data

Supplemental Figure Capion_V2.docx Supplemental Figure Capion_V2.docx

PGRN paper_ suplemetal Figure.pdf PGRN paper_ suplemetal Figure.pdf
