# Supplementary material for: Progranulin depletion inhibits proliferation via the transforming growth factor beta/SMAD family member 2 signaling axis in Kasumi-1 cells
Source: Heliyon. 2021 Jan 8;7(1):e05849. doi: 10.1016/j.heliyon.2020.e05849 (PMC7809376; doi:10.1016/j.heliyon.2020.e05849)
Supplement: Supplemental Figure Capion_V2.docx [file mmc1.docx]

**Title**

**Progranulin depletion inhibits proliferation via the transforming growth factor beta/SMAD family member 2 signaling axis in Kasumi-1 cells**

**Author information**

Kuniaki Yabe^a, b^, Yasuko Yamamoto^a,^ *, Masao Takemura^c^, Takeshi Hara^d^, Hisashi Tsurumi^d^, Ginette Serrero^e, f^, Toshitaka Nabeshima^c, g^, Kuniaki Saito^a, c, g, h^

**Supplemental Figure caption**

**Supplementary Figure. 1 Progranulin depletion inhibits Akt/mTOR pathway and proliferation of SLVL cells.**

SLVL cells transfected progranulin-specific siRNA or control siRNA were cultured with rapamycin (20 nM). Proliferation of treated cells was determined by MTT assay. Error bars indicate the SD from mean; *n* = 3. (*** *P* < 0.001 between Ctrl and PGRN KD, ꝉ *P* < 0.05 between Ctrl + rapamycin and PGRN KD + rapamycin; Tukey’s test)

**Supplementary Figure. 2 Progranulin depletion decreases phosphorylation level of ERK protein in Kasumi-1 cells.**

Kasumi-1 cells were cultured with anti progranulin antibody (200 μg/ml) or control antibody for 8 h. Whole cell lysate was collected and expression level of P-ERK and t-ERK protein was analyzed by Western blotting. Phosphorylation level of protein was normalized to that of total protein. Error bars indicate the SD from mean; *n* = 3. (** *P* < 0.01; two-tailed Student’s *t* test)

**Supplementary Figure. 3 Full, non-adjusted images of Western blotting.**

**a)** Original ECL image of anti-progranulin antibody blotted membrane, it is showed as Figure 1a. **b)** Original ECL image of anti-β-actin antibody blotted membrane, it is showed as Figure 1a. **c)** Original ECL image of anti-P-Akt antibody blotted membrane, it is shown as Figure 2a. **d)** Original ECL image of anti-t-Akt antibody blotted membrane, it is shown as Figure 2a. **e)** Original ECL image of anti-P-mTOR antibody blotted membrane, it is shown as Figure 2a. **f)** Original ECL image of anti-t-mTOR antibody blotted membrane, it is shown as Figure 2a. **g)** Original ECL image of anti-β-actin antibody blotted membrane, it is showed as Figure 2a. **h)** Original ECL image of anti-PARP antibody blotted membrane, it is showed as Figure 3a. **i)** Original ECL image of anti-caspase-3 antibody blotted membrane, it is showed as Figure 3a. **j)** Original ECL image of anti-Bax antibody blotted membrane, it is showed as Figure 3a. **k)** Original ECL image of anti-Bcl-2 antibody blotted membrane, it is showed as Figure 3a. **l)** Original ECL image of anti-XIAP antibody blotted membrane, it is showed as Figure 3a. **m)** Original ECL image of anti-β-actin antibody blotted membrane, it is showed as Figure 3a. **n)** Original ECL image of anti-PARP antibody blotted membrane, it is showed in the left side of Figure 3d. **o)** Original ECL image of anti-β-actin antibody blotted membrane, it is showed in the left side of Figure 3d. **p)** Original ECL image of anti-PARP antibody blotted membrane, it is showed in the right side of Figure 3d. **q)** Original ECL image of anti-β-actin antibody blotted membrane, it is showed in the right side of Figure 3d. **r)** Original ECL image of anti-TGF-β antibody blotted membrane, it is showed as Figure 4b. **s)** Original ECL image of anti-P-Smad2 antibody blotted membrane, it is showed as Figure 4b. **t)** Original ECL image of anti-t-Smad2 antibody blotted membrane, it is showed as Figure 4b. **u)** Original ECL image of anti-β-actin antibody blotted membrane, it is showed as Figure 4b. **v)** Original ECL image of anti-P-Smad3 antibody blotted membrane, it is showed as Figure 4e. **w)** Original ECL image of anti-t-Smad3 antibody blotted membrane, it is showed as Figure 4e. **x)** Original ECL image of anti-PARP antibody blotted membrane, it is showed as Figure 5a. **y)** Original ECL image of anti-β-actin antibody blotted membrane, it is showed as Figure 5a.
