# Supplementary material for: Progranulin depletion inhibits proliferation via the transforming growth factor beta/SMAD family member 2 signaling axis in Kasumi-1 cells
Source: Heliyon. 2021 Jan 8;7(1):e05849. doi: 10.1016/j.heliyon.2020.e05849 (PMC7809376; doi:10.1016/j.heliyon.2020.e05849)
Supplement: PGRN paper_ suplemetal Figure.pdf [file mmc2.pdf]

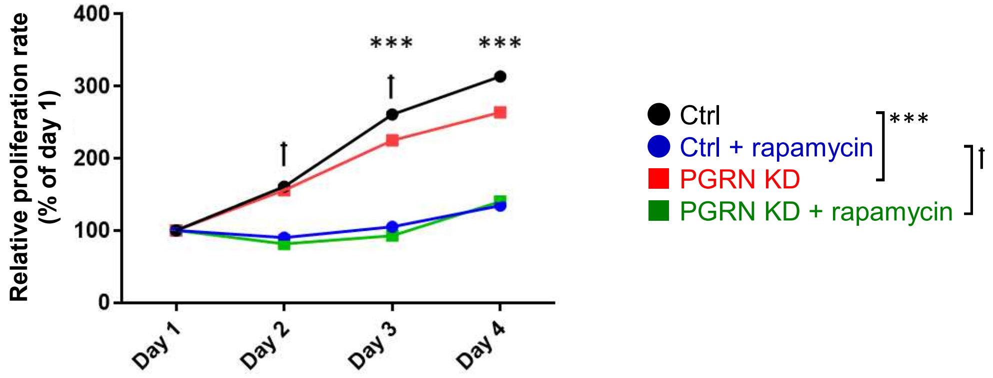

Supplementary Figure. 1

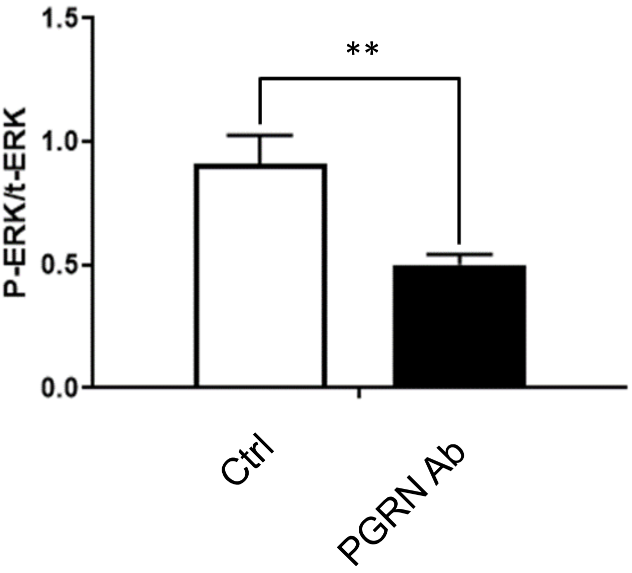

Supplementary Figure. 2

a)

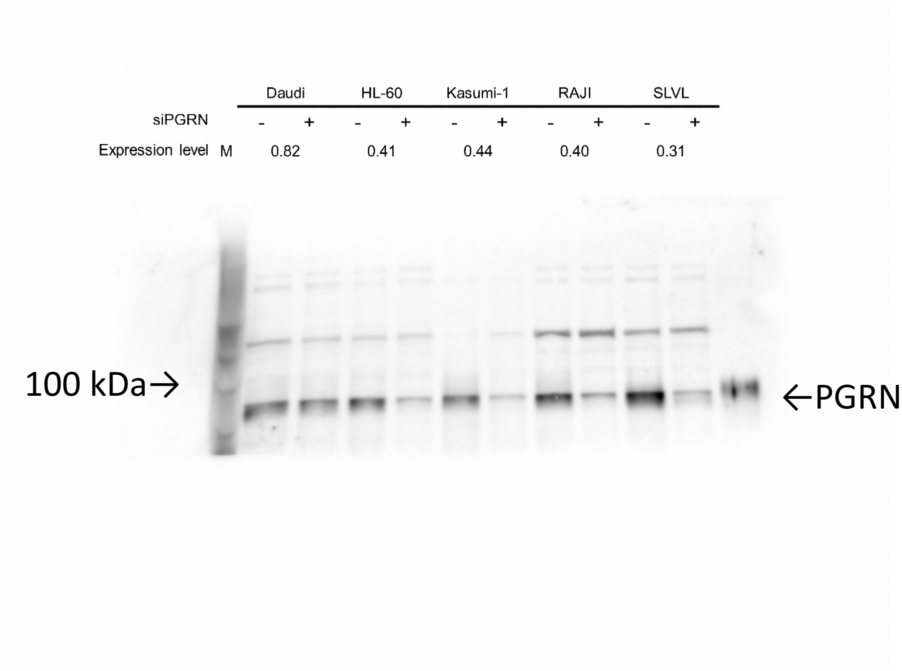

75 kDa→

b)

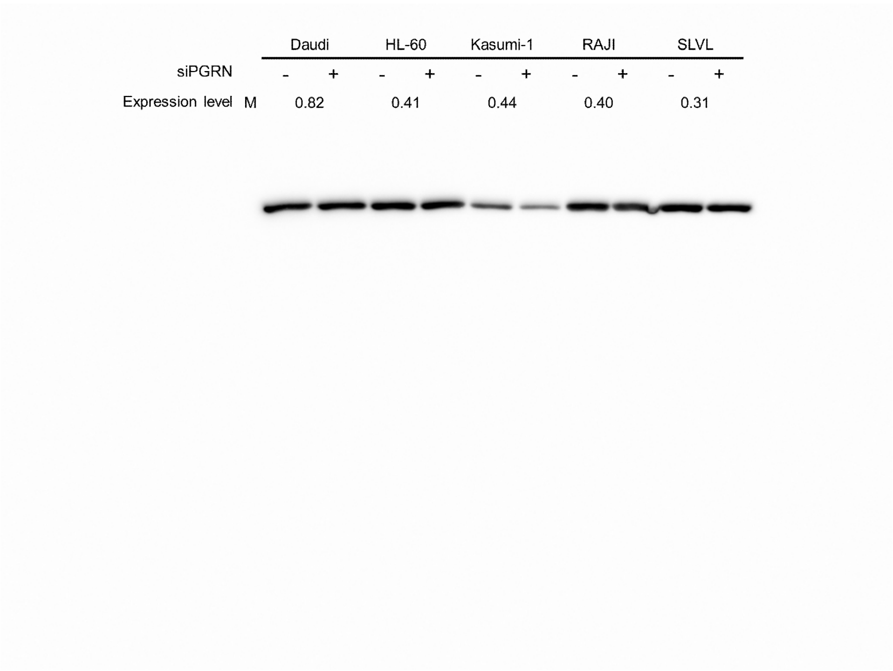

←β-actin

Supplementary Figure. 3

c)

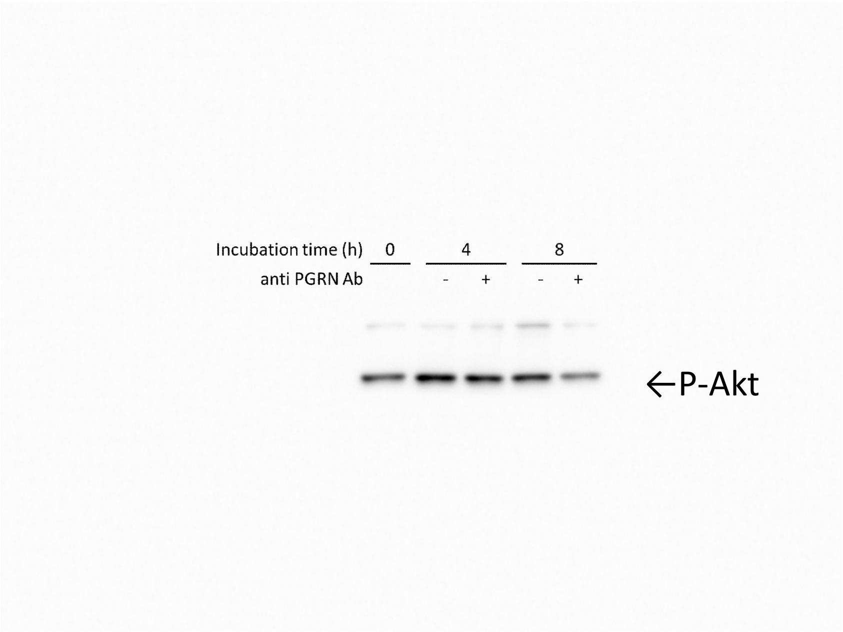

d)

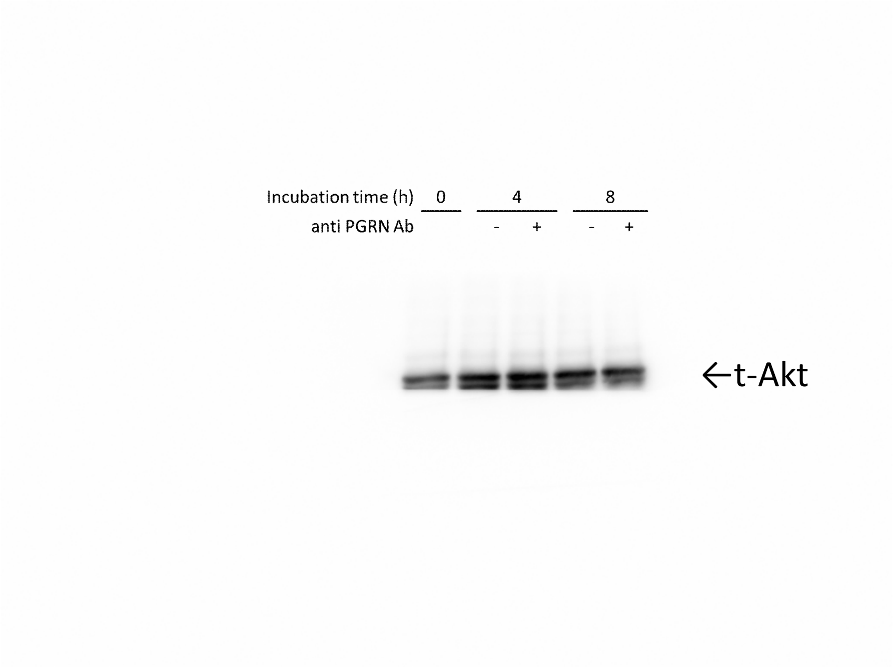

Supplementary Figure. 3

e)

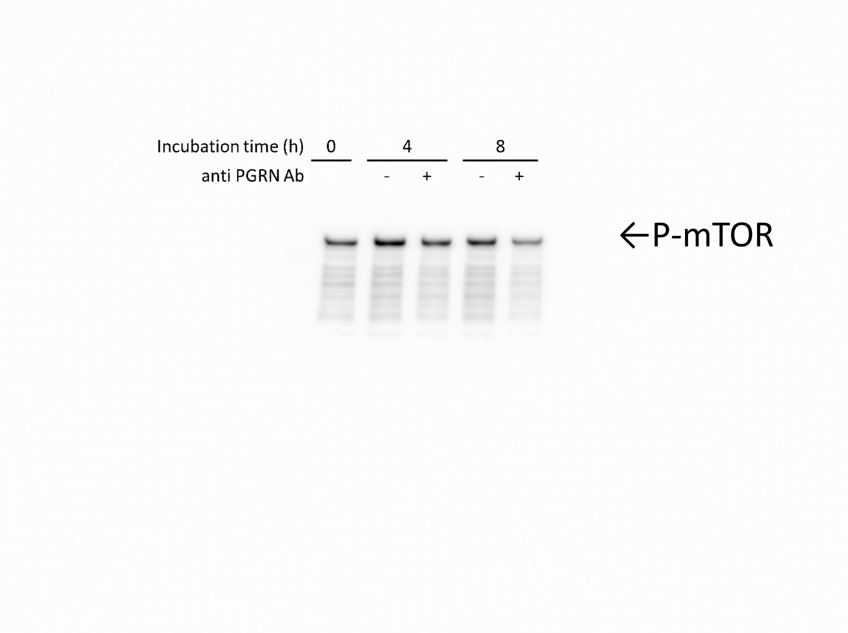

f)

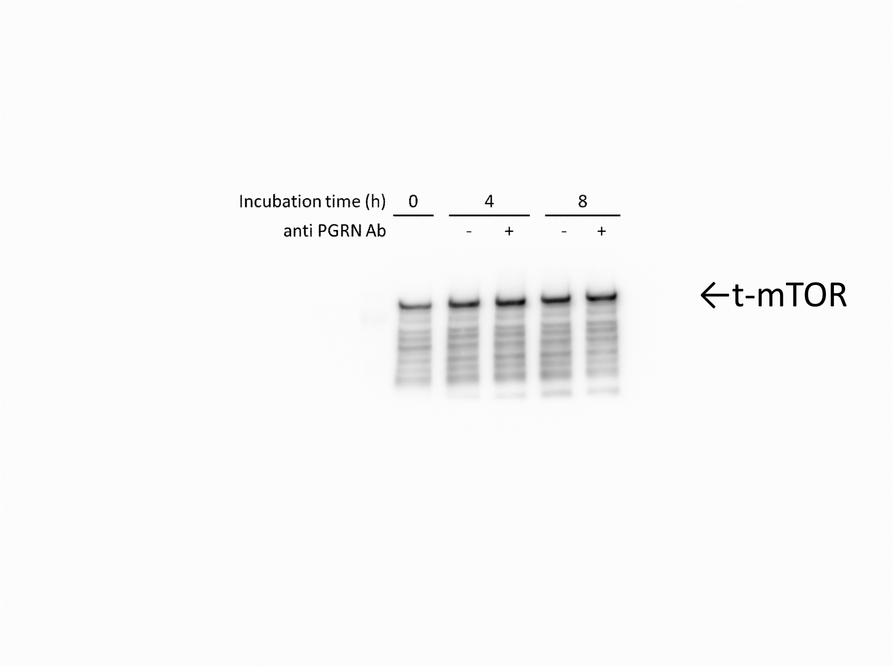

Supplementary Figure. 3

g)

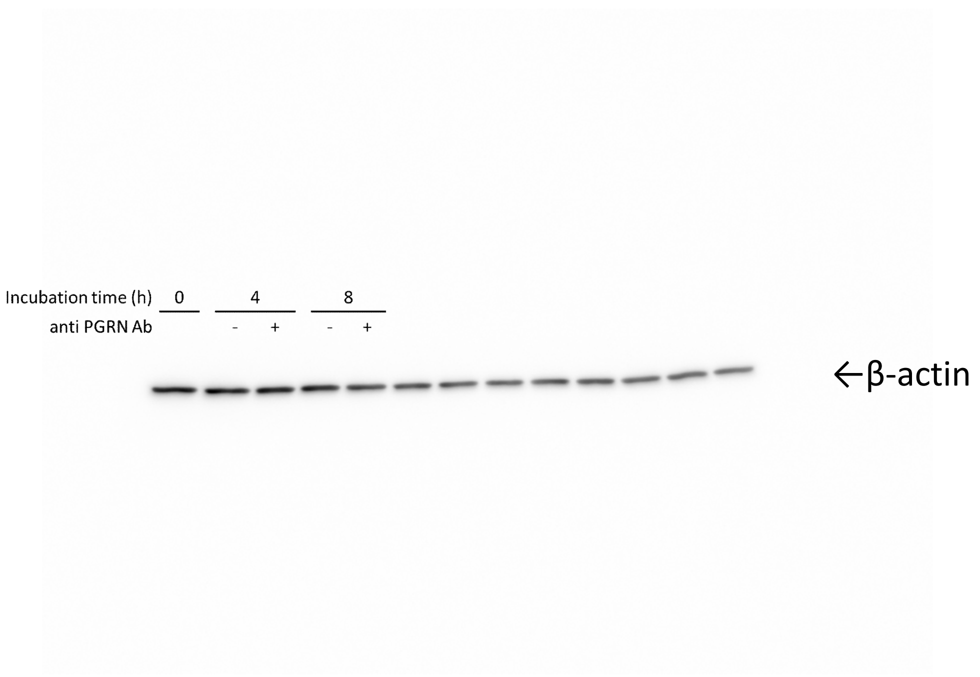

h)

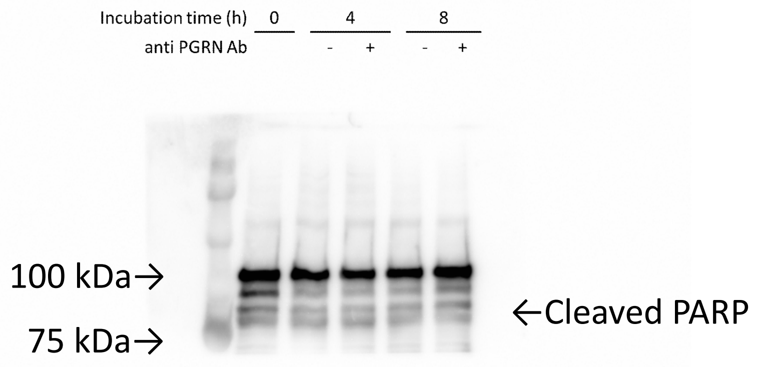

Supplementary Figure. 3

i)

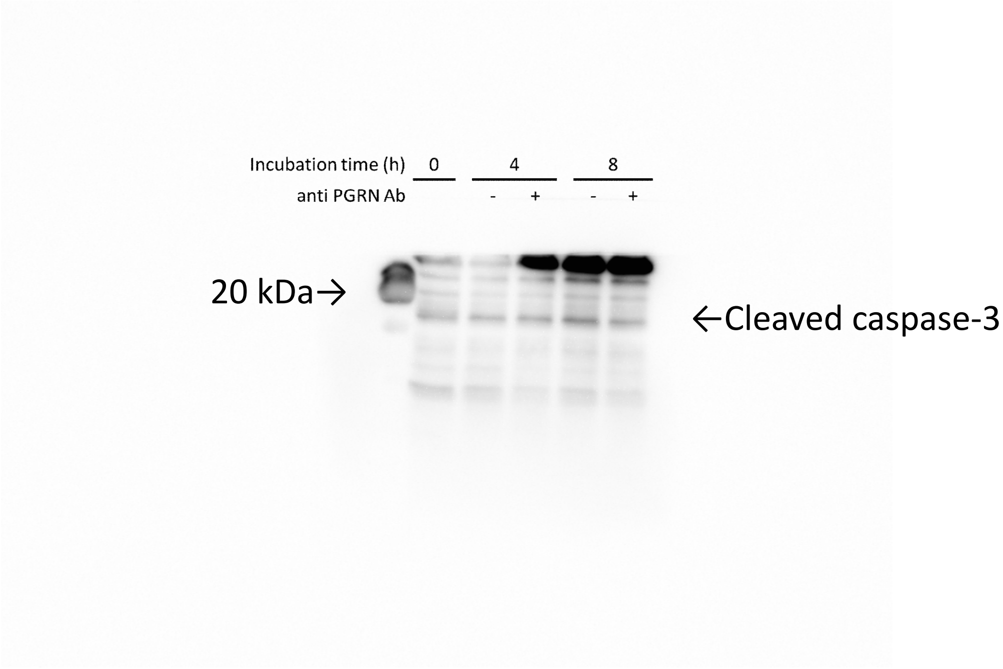

j)

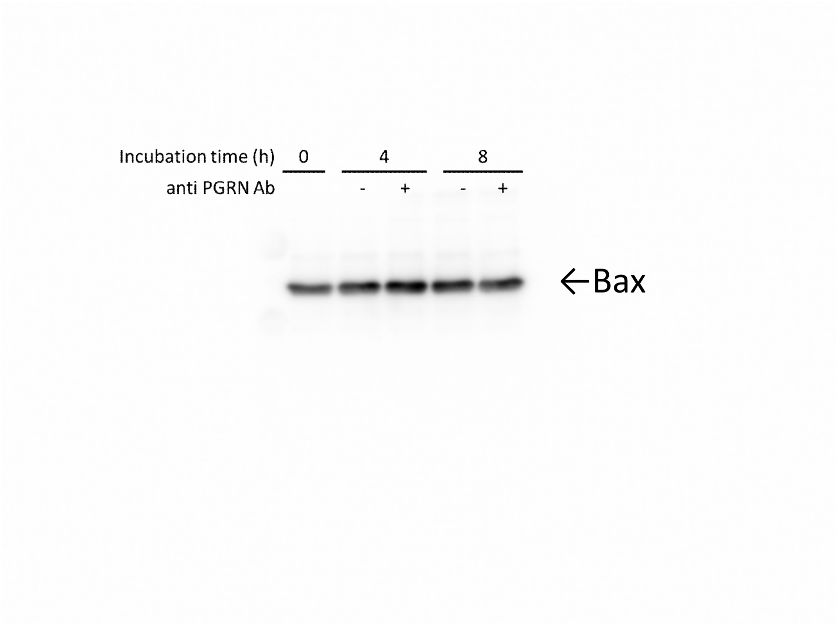

Supplementary Figure. 3

k)

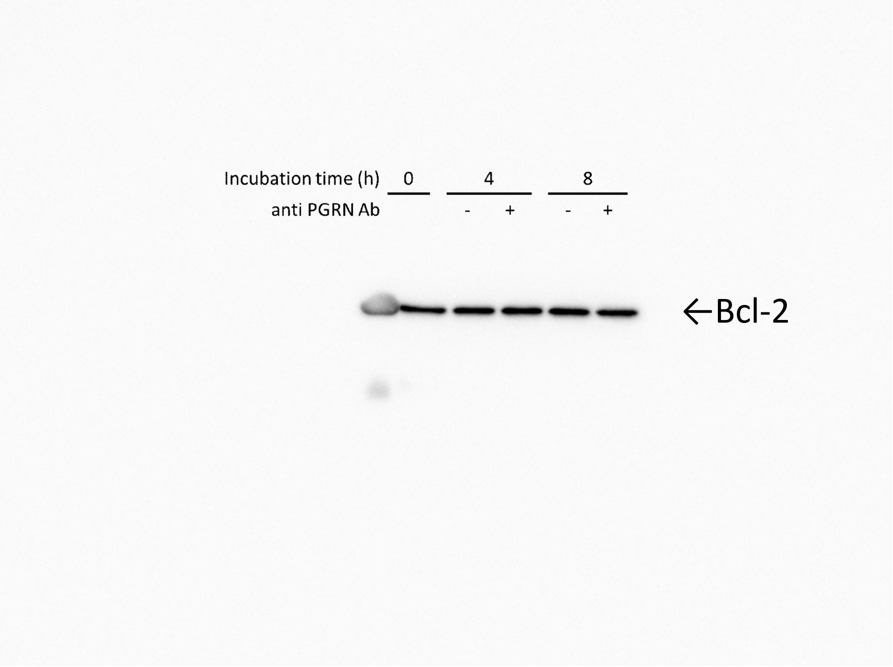

l)

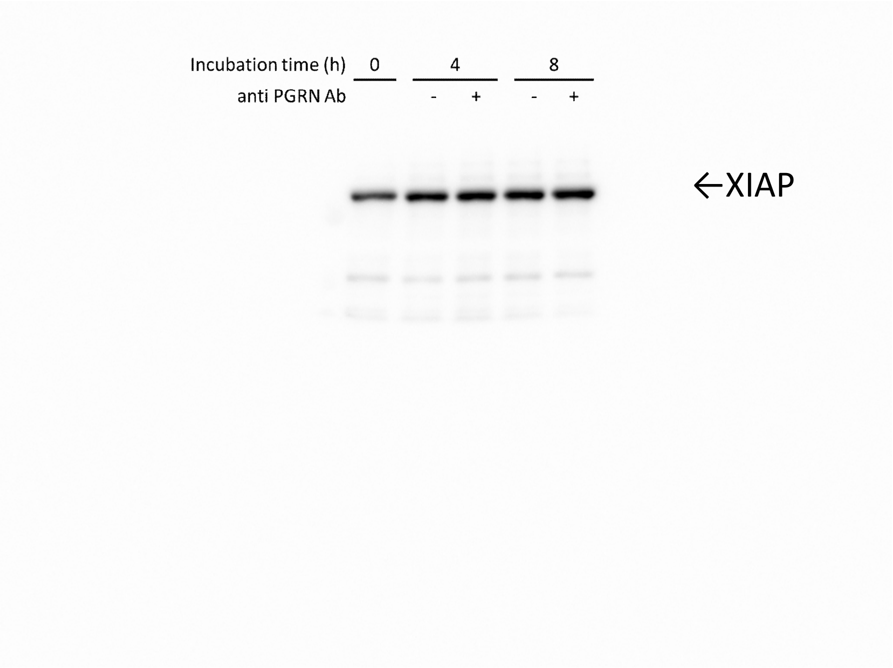

Supplementary Figure. 3

m)

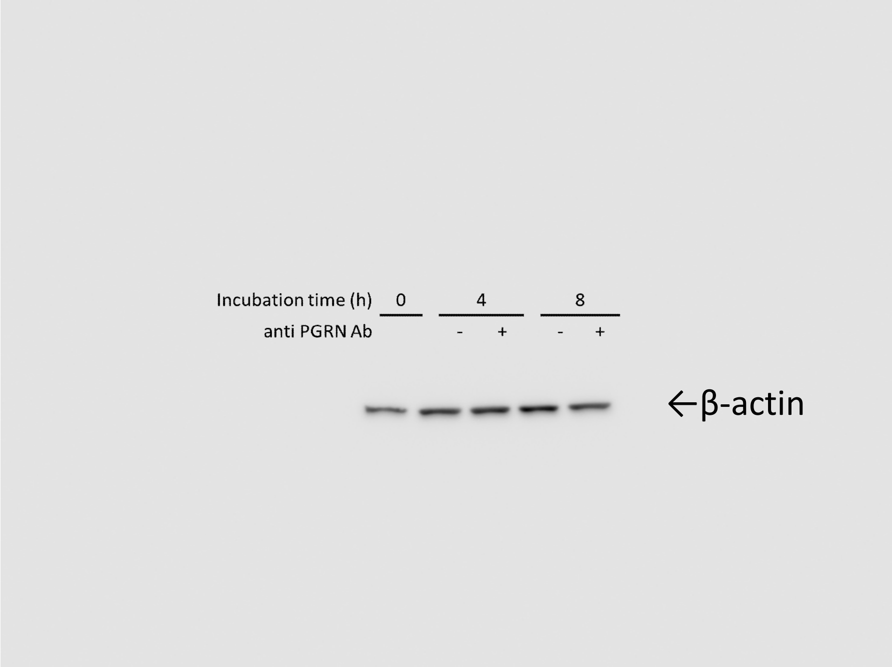

n)

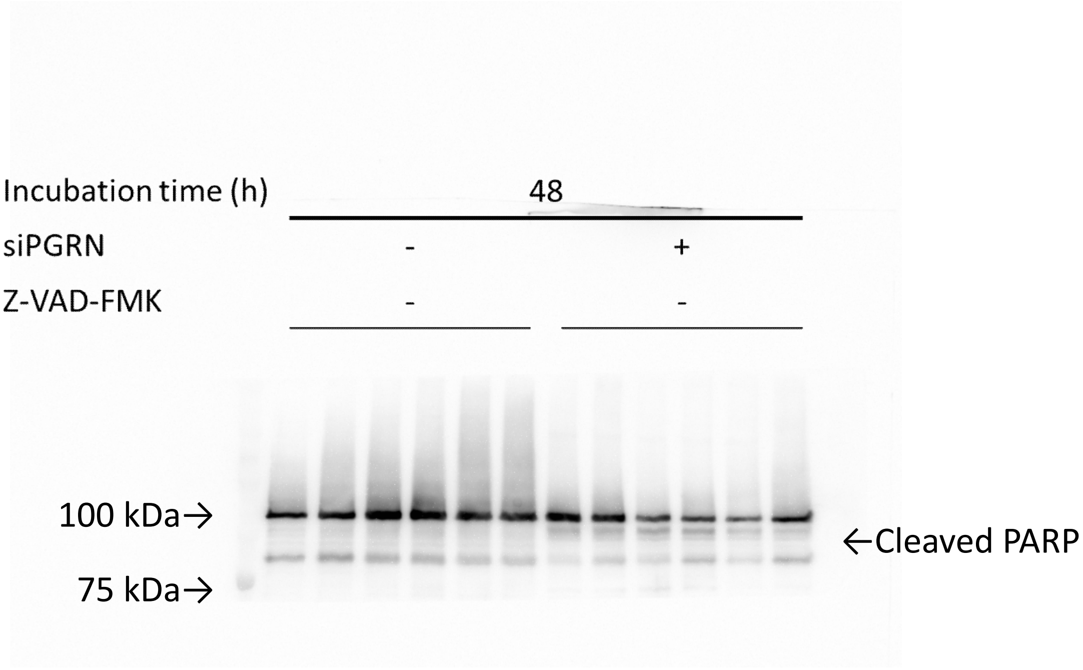

Supplementary Figure. 3

o)

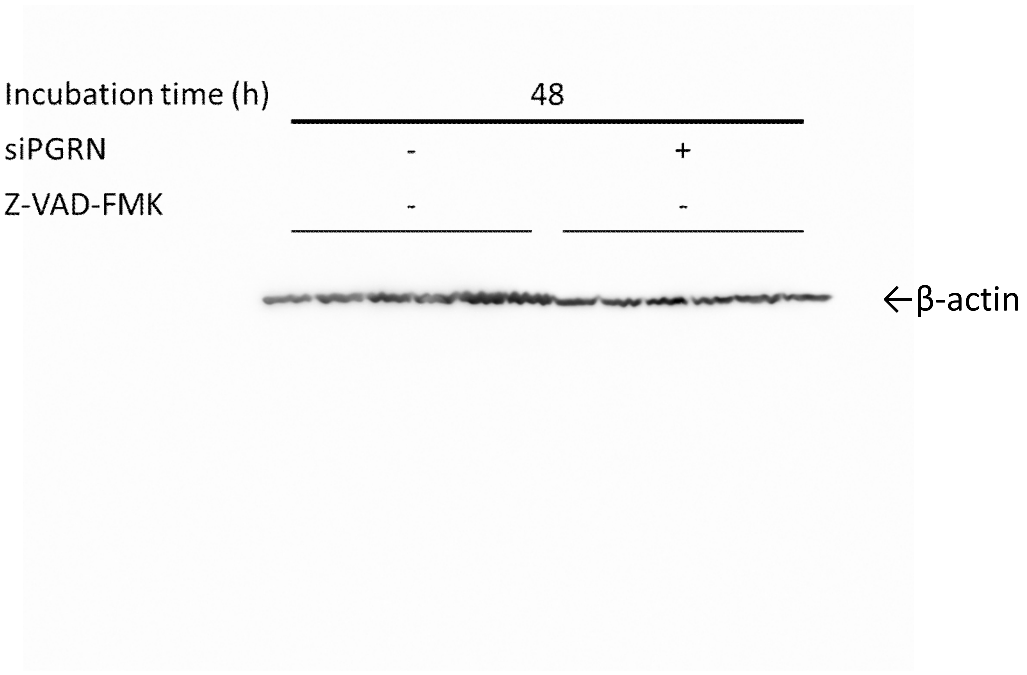

p)

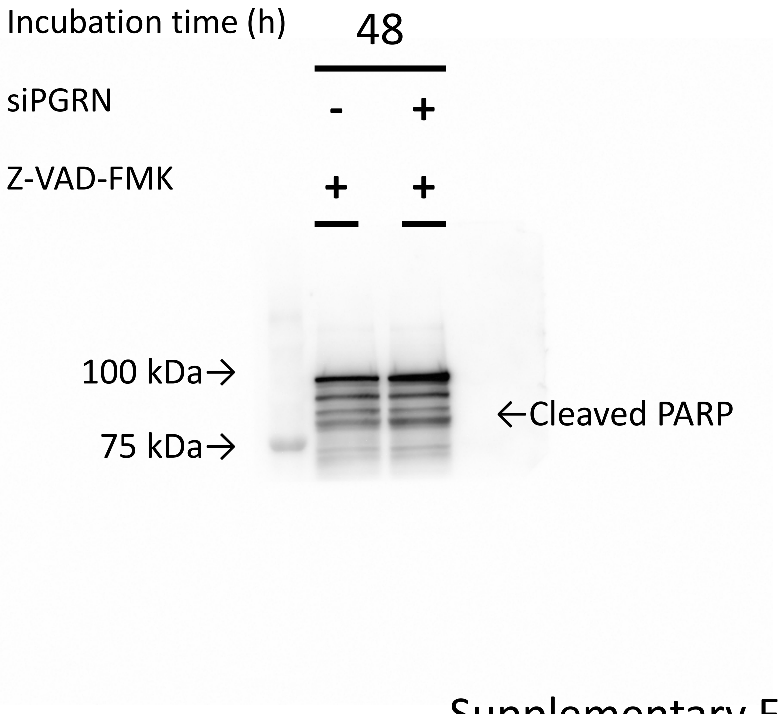

Supplementary Figure. 3

q)

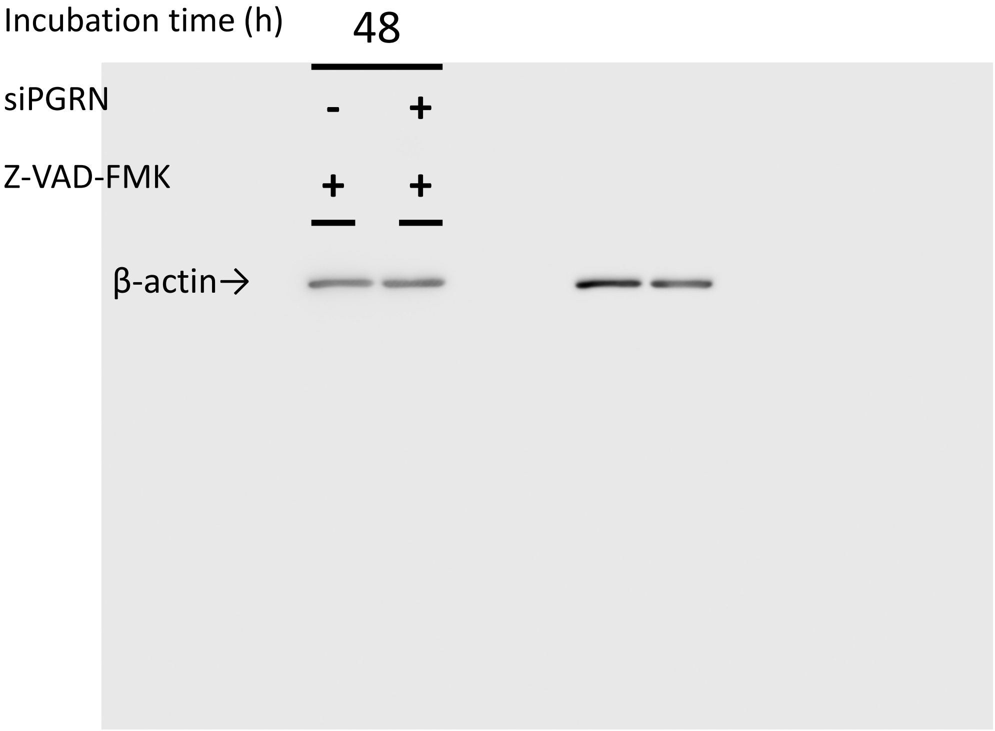

r)

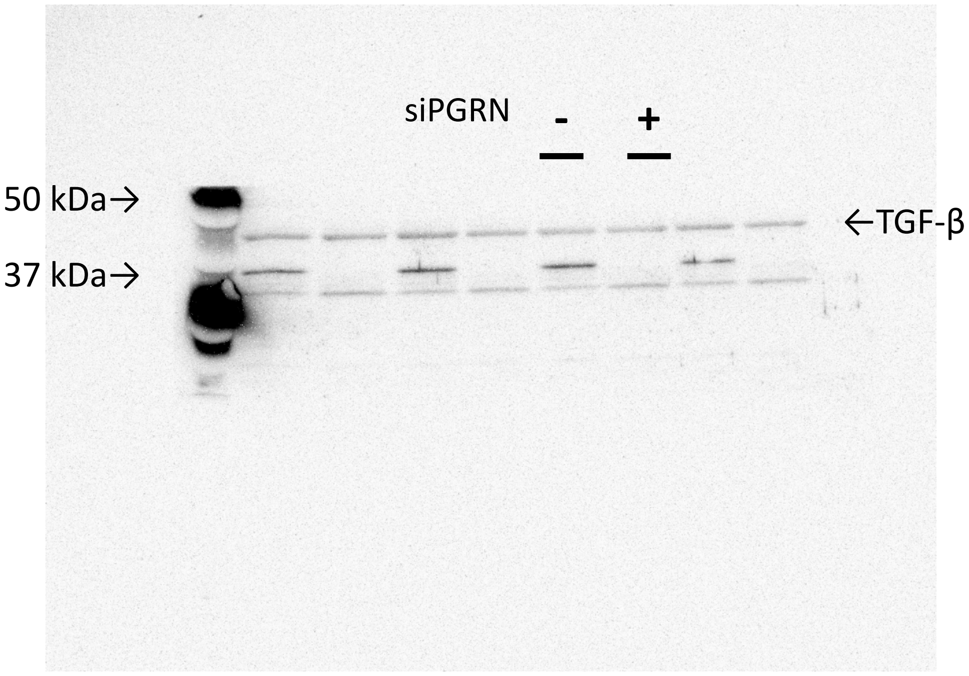

Supplementary Figure. 3

s)

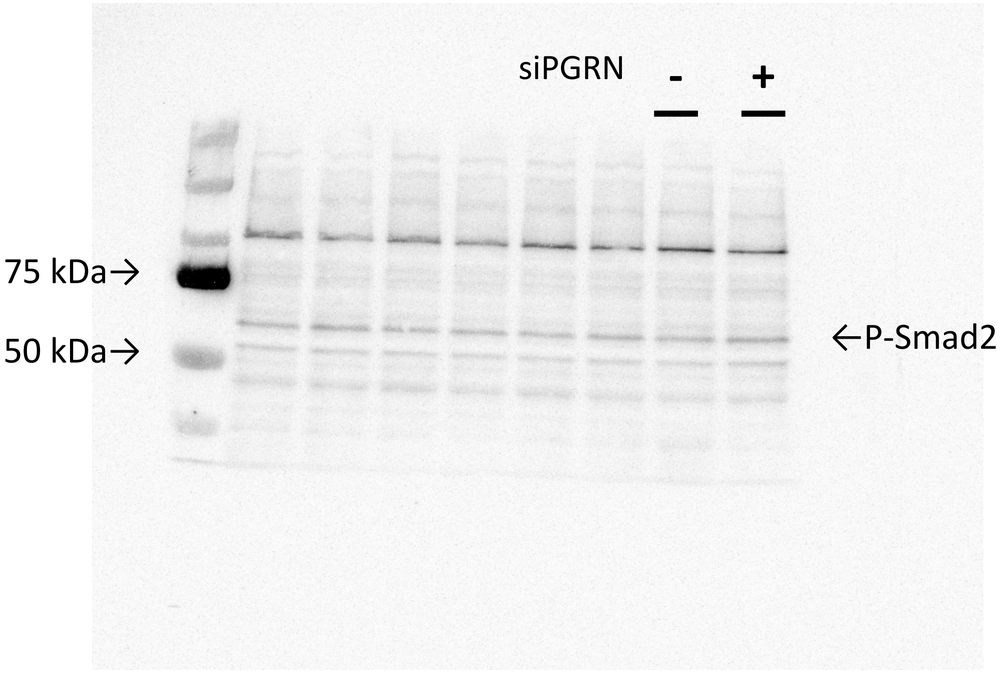

t)

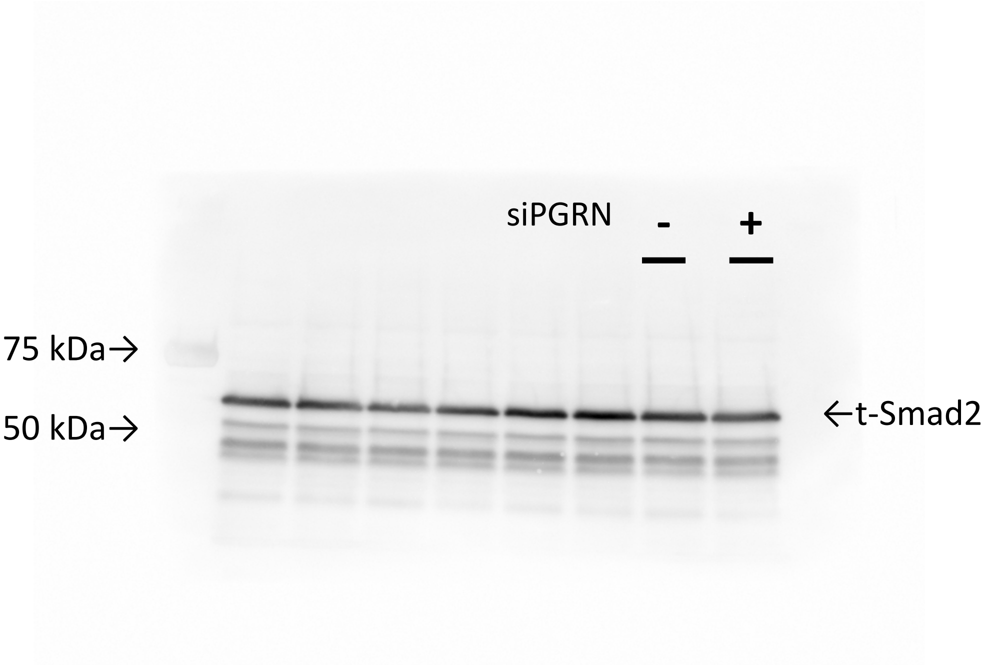

Supplementary Figure. 3

u)

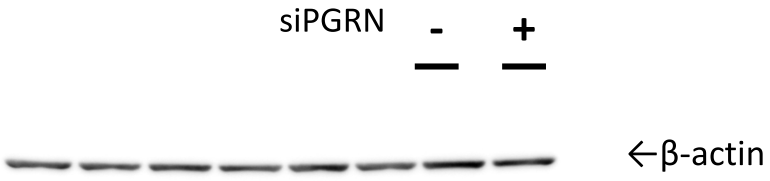

v)

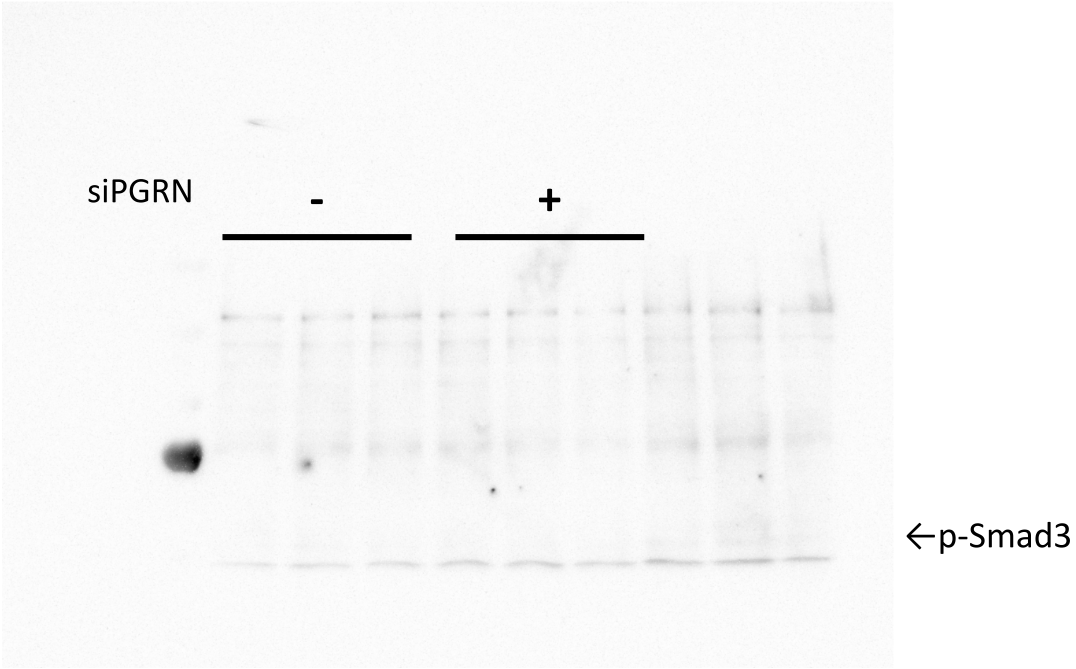

Supplementary Figure. 3

w)

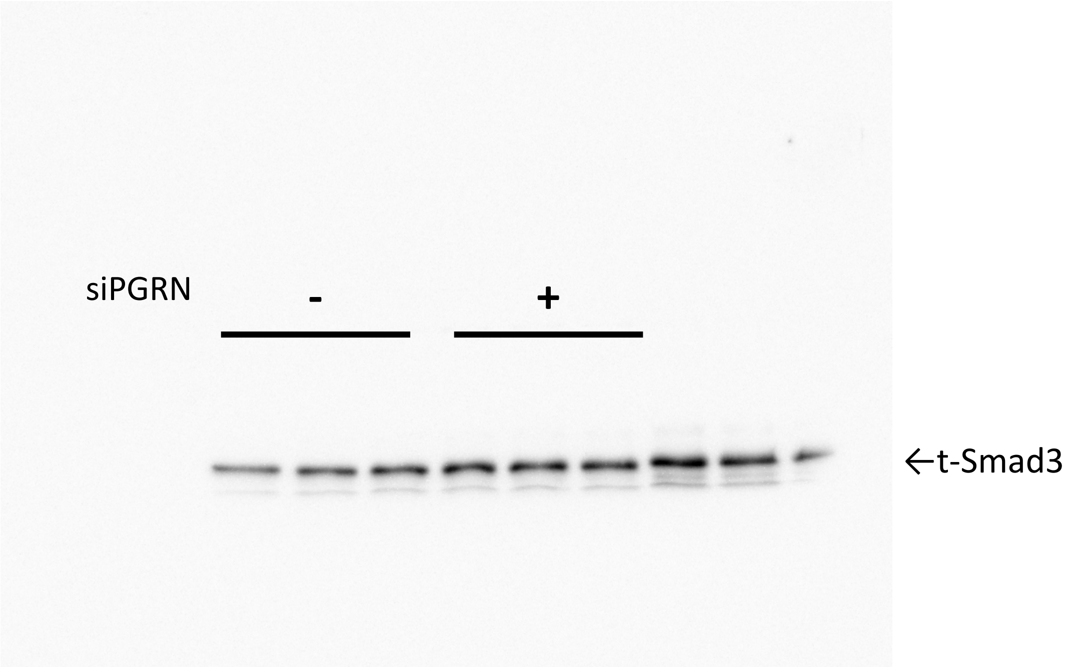

x)

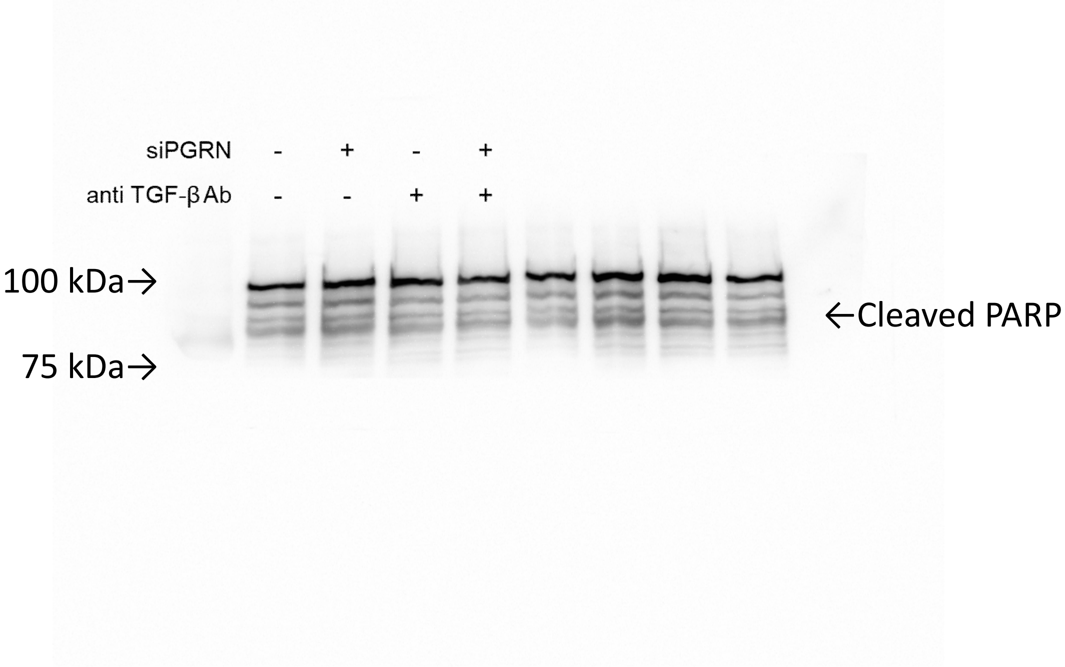

Supplementary Figure. 3

y)

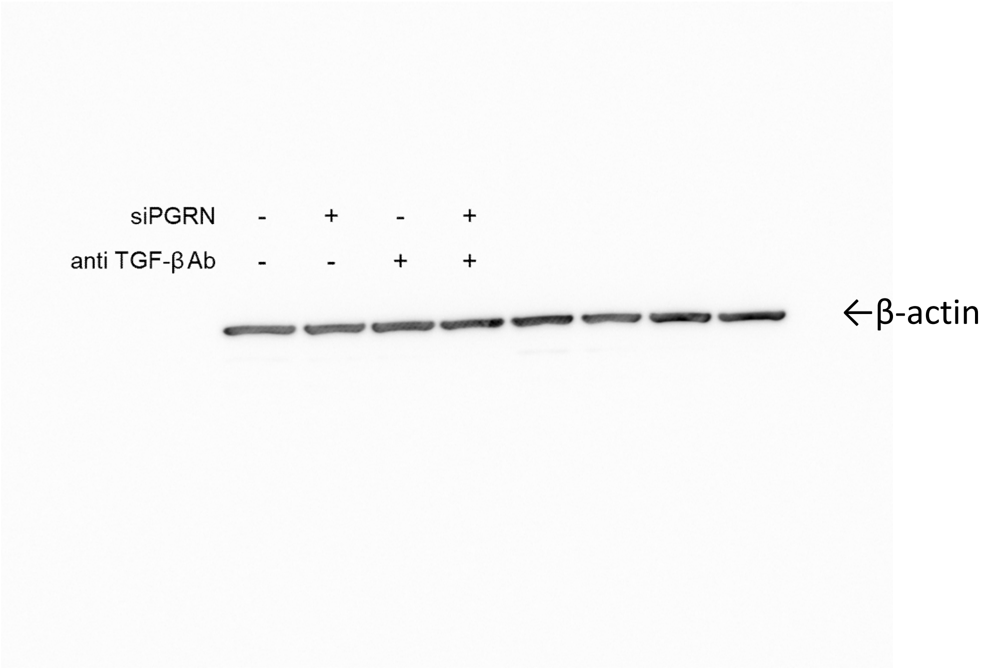

Supplementary Figure. 3
